# Identification of *Vicia* Species Native to South Korea Using Molecular and Morphological Characteristics

**DOI:** 10.3389/fpls.2021.608559

**Published:** 2021-02-09

**Authors:** Seahee Han, Raveendar Sebastin, XiaoHan Wang, Kyung Jun Lee, Gyu-Taek Cho, Do Yoon Hyun, Jong-Wook Chung

**Affiliations:** ^1^National Agrobiodiversity Center, National Institute of Agricultural Sciences, Rural Development Administration, Jeonju, South Korea; ^2^Department of Industrial Plant Science and Technology, Chungbuk National University, Cheongju, South Korea

**Keywords:** DNA barcoding, morphology, phylogeny, *Vicia* spp., species discrimination

## Abstract

Recently, within the Fabaceae family, the *Vicia* genus has been recognized for its vital role in sustainable agriculture. *Vicia* species are economically important grain and forage crops. However, the presence of complex morphological characteristics makes identification and recognition of native species difficult. In this study, the possibility of using DNA barcoding regions (ITS2, *matK*, and *rbcL*) to distinguish among 19 *Vicia* taxa (59 accessions) found in South Korea was evaluated. The sequence alignment analysis revealed considerable nucleotide diversity (π) between the loci, in which ITS2 showed the highest mean interspecific distance, whereas there was no intraspecific variability among the barcode regions in 12 of the 19 taxa. Phylogenetic analysis of combined barcoding regions revealed well-resolved phylogeny with the highest species level discrimination. Combinations of barcode loci were also used in classification at the subgenera and section levels. The results revealed that the combined barcoding regions can be used effectively to differentiate the following species: *Vicia angustifolia* var. *segetilis*, *Vicia bungei*, *Vicia villosa*, *Vicia cracca*, *Vicia dasycarpa*, *Vicia hirsuta*, *Vicia tetrasperma*, *Vicia amurensis*, *Vicia hirticalycina*, and *Vicia chosenensis*. However, it is difficult to differentiate the species of *Vicia unijuga*, *Vicia unijuga* var. *kaussanensis*, *Vicia linearifolia*, *Vicia unijuga* f. *angustifolia*, *Vicia nipponica*, *Vicia amoena*, *Vicia venosa* var. *cuspidata*, *Vicia pseudo-orobus*, and *Vicia japonica* with the tested barcode regions. These species come under sect. *Vicilla* and are found to be closely related or species that have recently undergone speciation; thus, it has limitation to distinguish with recommended barcodes. Hence, to differentiate the unclassified species, 39 morphological characteristics were investigated, in which 16 useful characteristics were selected for efficient classification. Finally, the 16 selected morphological useful traits efficiently differentiated all the *Vicia* species. In conclusion, a combination of barcoding loci together with morphological characteristics of this study efficiently discriminated all the Korean *Vicia* species.

## Introduction

The genus *Vicia* L. belongs to the Fabaceae (Leguminosae) family and is the third largest family of flowering plants worldwide. Approximately 150–210 species are distributed across Europe, Asia, and North America, and the majority of species is found in the Mediterranean (Cacan et al., 2016; Al-Joboury, 2017). To date, approximately 17–21 *Vicia* species have been reported in Korea (Lee, 1996; Choi, 2007; Nam et al., 2012). *Vicia* L. belongs to the Fabeae (syn. Vicieae) tribe along with *Lathyrus* L., *Lens* Mill., *Pisum* L., and *Vavilovia* Fed. (Kupicha, 1976). Furthermore, the *Vicia* genus is divided into two subgenera, *Vicia* L. (22 sections) and *Cracca* Peterm. [=*Vicilla* (SCHUR) ROUY, five sections], based on the presence of stipule nectaries, relative peduncle length, subtending leaves, and number of flowers per inflorescence (Kupicha, 1976; Leht, 2009).

Many Fabaceae species are economically important as crops used in food, herbal medicine, petroleum materials, and animal feeds (Koçak et al., 2011). The genus *Vicia* has the advantage of primarily containing dual-purpose crops (Francis et al., 2000). Representative *Vicia* species in agriculture are *Vicia villosa* Roth and *Vicia cracca* L., which have been used as green manure, cover, and forage crops. The ecological role of *Vicia* species such as *V. villosa* and *Vicia angustifolia* L. as hosts to nitrogen-fixing bacteria (*Rhizobium leguminosarum*) has the ability to fix free nitrogen from the air (Handi, 1982). It helps to fertilize the soil by the fixed nitrogen released and making it available to other plants in agricultural and natural systems. In South Korea, *Vicia* species are distributed in various habitats. For example, *Vicia tetrasperma* (L.) Schreb. and *Vicia hirsuta* (L.) Gray is widely distributed in range of grasslands, fields, cultivated areas, hill slopes, and roadsides, and *Vicia unijuga* A. Braun, *Vicia linearifolia* Y.N.Lee, *Vicia pseudo-orobus* Fisch. & C.A.Mey., and *Vicia nipponica* Matsum are mainly distributed in forests, mountain, and hill areas. *V. unijuga* var. *kaussanensis* H. Lév., *Vicia hirticalycina* Nakai, and *Vicia chosenensis* Ohwi are known as endemic plants of Korea, which have a restricted distribution with more threat to extinction than any other widely distributed species (Chung et al., 2017). Hence, there is an urgency of protecting the species to conserve the biological diversity. Recently, the agricultural utility and ecological roles of *Vicia* species have been recognized, and many studies on the genus are actively underway (Raveendar et al., 2015, 2017; Sveinsson and Cronk, 2016; Li et al., 2018; Wu et al., 2020).

Several candidate DNA regions in plastids, including *matK*, *rbcL*, and *trnH-psbA* spacers have been recommended for use in plant species identification (Kress and Erickson, 2007; Group et al., 2009). The *rbcL* and *matK* genes are well conserved in the plastid coding region of most plant species. They are most promising coding regions in chloroplasts for plant species identification, with low transition and transversion rates. Similarly, due to the high level of sequence divergence, *trnH-psbA* spacers region has been considered as the best candidate plant barcode (Hollingsworth et al., 2011). However, species discrimination has previously been impossible when using single DNA barcodes for species identification. Hence, the Consortium for the Barcode of Life Plant Working Group suggested seven candidate plastid DNA regions (*atpF-atpH* spacer, *matK* gene, *rbcL* gene, *rpoB* gene, *rpoC1* gene, *psbK-psbI* spacer, and *trnH-psbA* spacer) and recommended the two-locus combination of *rbcL* + *matK* as a standard plant barcode (Group et al., 2009; Hollingsworth et al., 2011). In addition, combining nuclear [internal transcribed spacer (ITS)] and plastid (*matK*, *rbcL*, and *psbA-trnH*) regions has been recognized as a potential method of classifying various plant species (Chen et al., 2010; Gao et al., 2010; Purty and Chatterjee, 2016). In eukaryotes, the ITS region is present in the nuclear 45S ribosomal RNA (rDNA) genes, which can be further divided into the ITS1 and ITS2 regions. It has been proven to be the most suitable area for DNA barcoding, as it has a high efficiency of PCR amplification (93.8%) in most plant species (Chen et al., 2010). The use of multiple DNA barcodes may provide sufficient identification power for closely related species (Cold Spring Harbor DNA Learning Center, 2014).

Similarly, morphological markers have been studied in combination with molecular markers as an efficient method of species classification (Carneiro de Melo Moura et al., 2019; Xie et al., 2019; Martínez-Arce et al., 2020). Morphological data always play a key role in plant species classification based on phylogeny (Scotland et al., 2003). However, morphological studies require in-depth knowledge of characteristics or the number of species. Molecular tools using DNA barcodes for taxonomic identification, species delimitation, and access to phylogeny have the potential to overcome these difficulties faced by taxonomists (Martínez-Arce et al., 2020). However, it is very difficult to resolve recently diverged species or new species generated through hybridization if there is no universal sequence of common region as DNA barcode, and sufficient sequence divergence preserved in the domains of all living organisms is necessary for species classification (Ali et al., 2014). DNA barcoding can be used together with traditional morphology, and it is necessary to use data derived from morphology in tandem with DNA sequence data (Wen and Pandey, 2005; Ali and Choudhary, 2011; Ali et al., 2014). Thus, integrating DNA sequencing, morphology, and ecology would lead to successful species classification (Dasmahapatra and Mallet, 2006).

Morphological and DNA barcoding studies have been carried out to discriminate among *Vicia* species. In terms of morphology, style shape, stipule margin, and size, tendril, leaflets, flower length, peduncles, presence/absence of stipule nectaries, calyx teeth, hairs, legume shape, and ovary characteristics are known to be important for species identification (Kupicha, 1976; Jalilian et al., 2014). Karyological studies using chromosomes, pollen morphology, characters, and testa texture of seeds have also been carried out in *Vicia*, and these traits have been recognized as important taxonomic keys (Maxted et al., 1991; Chernoff et al., 1992; Endo and Ohashi, 1995; Nam et al., 2012). However, most of these taxonomic traits require specialized taxonomic knowledge and experience, and vast quantities of morphological information are required at each plant growth stage.

The ITS region of the nuclear genome was initially recommended for use in identifying *Vicia* species (Choi et al., 2006; Endo et al., 2008; Shiran et al., 2014). Furthermore, the efficiency of species identification has been evaluated using different plastid barcode regions (Raveendar et al., 2017), and 161 *Vicia* species have been identified using a combination of barcoding loci such as *matK*, *rbcL*, *trnH-psbA*, *trnL-trnF*, ITS1, and ITS2 (Wu et al., 2020). Relationships analysis within the *Vicia* genus has revealed 10 sections, including one composed of East Asian species found in Korea, using molecular and stylar features to create the phylogeny (Choi et al., 2006). However, only the ITS region has been used in barcoding, and there has been no research into the morphological features of the 19 *Vicia* taxa distributed in South Korea. Therefore, the aim of the present study was to confirm the suitability of various barcoding (ITS2, *matK*, and *rbcL*) regions together with morphological traits for the identification of Korean *Vicia* species.

## Materials and Methods

### Plant Materials and DNA Extraction

**FIGURE 1:**
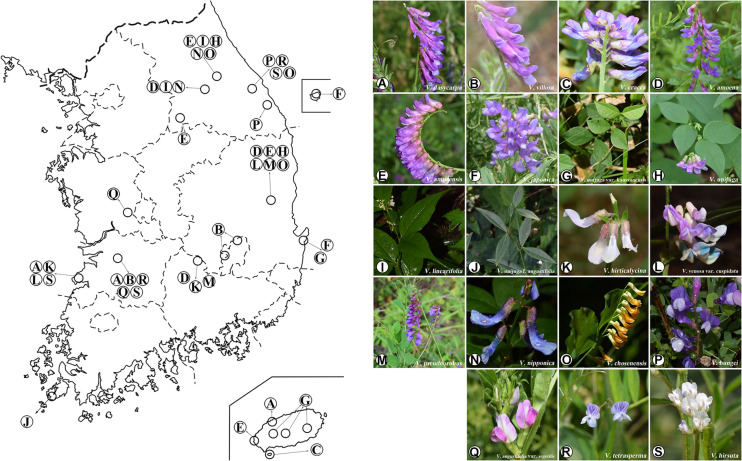
Collection sites of *Vicia* species in Korea. **(A)** *V. dasycarpa*; **(B)** *V. villosa*; **(C)** *V. cracca*; **(D)** *V. amoena*; **(E)** *V. amurensis*; **(F)** *V. japonica*; **(G)** *V. unijuga* var. *kaussanensis*; **(H)** *V. unijuga*; **(I)** *V. linearifolia*; **(J)** *V. unijuga* f. *angustifolia*; **(K)** *V. hirticalycina*; **(L)** *V. venosa* var. *cuspidata*; **(M)** *V. pseudo-orobus*; **(N)** *V. nipponica*; **(O)** *V. chosenensis*; **(P)** *V. bungei*; **(Q)** *V. angustifolia* var. *segetilis*; **(R)** *V. tetrasperma*; **(S)** *V. hirsuta*.

Samples of 19 *Vicia* taxa were collected from 59 different locations in South Korea (Figure 1 and Table 1). Voucher specimens were deposited to the Herbarium Conservation Center of the National Institute of Agricultural Sciences (Table 1). Barcode sequence of ITS2, *matK*, and *rbcL* for 33 *Vicia* species (about 88 accessions) conserved at the National Agrobiodiversity Center, South Korea were downloaded from NCBI GenBank. These data were combined with the present study to increase the efficient conservation and utilization of *Vicia* accessions in South Korea (Supplementary Table S1). Genomic DNA (gDNA) was extracted using DNeasy^®^ Plant Mini kits (Qiagen, Hilden, Germany) according to the manufacturer’s instructions. Freeze-dried leaves were used for DNA extraction and were ground into a powder. DNA was resuspended in 100 ml water, and dilutions were made up to 10 ng μl^–1^, after which samples were stored at −20°C. DNA quality and quantity were measured using 1% (w/v) agarose gel and a spectrophotometer (Epoch, BioTek, Winooski, VT, United States).

**TABLE 1:** List of plant species used in this study along with the collection site, GPS source, and voucher number.

No.	Scientific name	Subgenus	Section	References	Collection date	Collection site	GPS source	Voucher	ITS2	*matK*	*rbcl*
1	*V. dasycarpa* Ten.	*Cracca*	*Cracca*	Jalilian et al., 2014	180412	Jeju, Jeju-do, South Korea	N 33° 27′ 25.9″ E 126° 20′ 44.6″	KVA_RDA-2018-029	MW374737	MW372935	MW372994
2					180602	Buan, Jeonbuk, South Korea	N 35° 40′ 38.82″ E 126° 34′ 6.39″	KVA_RDA-2018-030	MW374738	MW372936	MW372995
3					180711	Jeonju, Jeonbuk, South Korea	N 35° 50′ 3.62″ E 127° 4′ 4.29″	KVA_RDA-2018-031	MW374739	MW372937	MW372996
4					180517	Jeonju, Jeonbuk, South Korea	N 35° 49′ 58.32″ E 127° 3′ 46.7	KVA_RDA-2018-032	MW374740	MW372938	MW372997
5					180516	Jeju, Jeju-do, South Korea	N 33° 27′ 25.9″ E 126° 20′ 34.6″	KVA_RDA-2018-033	MW374741	MW372939	MW372998
6	*V. villosa* Roth	*Cracca*	*Cracca*	Kupicha, 1976; Leht, 2009	180517	Jeonju, Jeonbuk, South Korea	N 35° 50′ 31.5″ E 127° 06′ 23.0″	KVA_RDA-2018-034	MW374742	MW372940	MW372999
7					170805	Jeonju, Jeonbuk, South Korea	N 35° 50′ 18.5″ E 127° 06′ 57.0″	KVA_RDA-2017-001	MW374743	MW372941	MW373000
8					180603	Daegu, Gyeongbuk, South Korea	N 35° 49′ 43.4″ E 128° 28′ 54.2″	KVA_RDA-2018-035	MW374744	MW372942	MW373001
9	*V. cracca* L.	*Cracca*	*Cracca*	Chernoff et al., 1992; Leht, 2009	180713	Jeju, Jeju-do, South Korea	N 33° 10′ 14.87″ E 126° 16′ 38.49″	KVA_RDA-2018-036	MW374745	MW372943	MW373002
10					180713	Jeju, Jeju-do, South Korea	N 33° 10′ 3.1″ E 126° 16′ 28.8′	KVA_RDA-2018-037	MW374746	MW372944	MW373003
11	*V. amoena* Fisch. ex DC.	*Cracca*	*Vicilla*	Kupicha, 1976; Leht, 2009	170810	Hoengseong, Gangwon, South Korea	N 37° 35′ 49.5″ E 128° 16′ 45.7′	KVA_RDA-2017-002	MW374747	MW372945	MW373004
12					170901	Hapcheon, Gyeongnam, South Korea	N 35° 46′ 32.7″ E 128° 07′ 31.5″	KVA_RDA-2017-003	MW374748	MW372946	MW373005
13					180907	Cheongsong, Gyeongbuk, South Korea	N 36° 24′ 41.6″ E 129° 10′ 52.83″	KVA_RDA-2018-038	MW374749	MW372947	MW373006
14	*V. amurensis* Oett.	*Cracca*	*Vicilla*	Kupicha, 1976; Leht, 2009	180526	Wonju, Gangwon, South Korea	N 37° 17′ 21.9″ E 128° 00′ 59.7″	KVA_RDA-2018-039	MW374750	MW372948	MW373007
15					170810	Hoengseong, Gangwon, South Korea	N 37° 35′ 17.7″ E 128° 16′ 54.3″	KVA_RDA-2017-004	MW374751	MW372949	MW373008
16					170820	Cheongsong, Gyeongbuk, South Korea	N 36° 22′ 14.37″ E 129° 9′ 38.5″	KVA_RDA-2017-005	MW374752	MW372950	MW373009
17					170714	Gapyeong, Gyeonggi, South Korea	N 37° 43′ 26.8″ E 127°, 27′, 38.4″	KVA_RDA-2017-006	MW374753	MW372951	MW373010
18					180526	Wonju, Gangwon, South Korea	N 37° 22′ 46.7″ E 127° 56′ 37.3	KVA_RDA-2018-040	MW374754	MW372952	MW373011
19					180803	Pyengchang, Gangwon, South Korea	N 37° 35′ 13.3″ E 128° 17′ 13.3″	KVA_RDA-2018-041	MW374755	MW372953	MW373012
20					180712	Jeju, Jeju-do, South Korea	N 33° 17′ 42.9″ E 126° 09′ 50.5″	KVA_RDA-2018-042	MW374756	MW372954	MW373013
21	*V. japonica* A. Gray	*Cracca*	*Vicilla*	Choi et al., 2006	171021	Pohang, Gyeongbuk, South Korea	N 35° 56′ 18.9″ E 129° 31′ 41.1″	KVA_RDA-2017-007	MW374757	MW372955	MW373014
22					170810	Ulleng, Gyeongbuk, South Korea	N 33° 22′ 2.68″ E 126° 20′ 39.2″	KVA_RDA-2017-008	MW374758	MW372956	MW373015
23					170810	Ulleng, Gyeongbuk, South Korea	N 37° 32′ 0.4″ E 130° 50′ 31.1″	KVA_RDA-2017-009	MW374759	MW372957	MW373016
24	*V. unijuga* var. *kaussanensis* H. Lév.	*Cracca*	*Vicilla*	Kupicha, 1976; Leht, 2009	171021	Pohang, Gyeongbuk, South Korea	N 35° 49′ 53.9″ E 127° 4 36.04	KVA_RDA-2017-010	MW374760	MW372958	MW373017
25					171030	Jeju, Jeju-do, South Korea	N 35° 49′ 53.9″ E 127° 4 36.04	KVA_RDA-2017-011	MW374761	MW372959	MW373018
26					180713	Jeju, Jeju-do, South Korea	N 33° 21′ 32.59″ E 126° 29′ 55.3″	KVA_RDA-2018-043	MW374762	MW372960	MW373019
27					180713	Jeju, Jeju-do, South Korea	N 33° 26′ 20.5″ E 126° 47′ 32.9″	KVA_RDA-2018-044	MW374763	MW372961	MW373020
28	*V. unijuga* A.Braun	*Cracca*	*Vicilla*	Kupicha, 1976; Leht, 2009	170826	Pyengchang, Gangwon, South Korea	N 37° 35′ 41.8″ E 128° 17′ 32.41″	KVA_RDA-2017-012	MW374764	MW372962	MW373021
29					170820	Cheongsong, Gyeongbuk, South Korea	N 36° 24′ 25.79″ E 129° 10′ 19.1″	KVA_RDA-2017-013	MW374765	MW372963	MW373022
30					180907	Cheongsong, Gyeongbuk, South Korea	N 36° 24′ 51.32″ E 129° 11′ 24.06″	KVA_RDA-2018-045	MW374766	MW372964	MW373023
31	*V. linearifolia* Y.N.Lee	*Cracca*	*Vicilla*	Choi et al., 2006	170810	Hoengseong, Gangwon, South Korea	N 37° 43′ 02.2″ E 128° 26′ 54.7″	KVA_RDA-2017-014	MW374767	MW372965	MW373024
32					180831	Hoengseong, Gangwon, South Korea	N 37° 42′ 50.3″ E 128°26′ 49.3″	KVA_RDA-2018-046	MW374768	MW372966	MW373025
33					170811	Hongcheon, Gangwon, South Korea	N 37° 42′ 50.3″ E 128°26′ 49.3″	KVA_RDA-2017-015	MW374769	MW372967	MW373026
34	*V. unijuga* f. *angustifolia* Makino ex Ohwi	*Cracca*	*Vicilla*	Kupicha, 1976; Leht, 2009	171009	Jindo, Jeonnam, South Korea	N 34° 22′ 17.67″ E 126° 9′ 27.8″	KVA_RDA-2017-016	MW374770	MW372968	MW373027
35					171009	Jindo, Jeonnam, South Korea	N 34° 21′ 37.36″ E 126° 10′ 4.87″	KVA_RDA-2017-017	MW374771	MW372969	MW373028
36	*V. hirticalycina* Nakai	*Cracca*	*Vicilla*	Choi et al., 2006	170514	Hapcheon, Gyeongnam, South Korea	N 35° 48′ 11.4″ E 128° 06′ 11.3″	KVA_RDA-2017-018	MW374772	MW372970	MW373029
37					180804	Buan, Jeonbuk, South Korea	N 35° 37′ 44.17″ E 126° 34′ 24.29	KVA_RDA-2018-047	MW374773	MW372971	MW373030
38					180616	Buan, Jeonbuk, South Korea	N 35° 38′ 14.2″ E 126° 34′ 44.7″	KVA_RDA-2018-048	MW374774	MW372972	MW373031
39	*V. venosa* var. *cuspidata* Maxim.	*Cracca*	*Vicilla*	Kupicha, 1976; Leht, 2009	180616	Buan, Jeonbuk, South Korea	N 35° 41′ 53.87″ E 126° 36′ 11.89″	KVA_RDA-2018-049	MW374775	MW372973	MW373032
40					180907	Cheongsong, Gyeongbuk, South Korea	N 35° 39′ 42.5″ E 126° 39′ 0.1″	KVA_RDA-2018-050	MW374776	MW372974	MW373033
41					180930	Buan, Jeonbuk, South Korea	N 36° 26′ 26.31″ E 129° 8 8.14	KVA_RDA-2018-051	MW374777	MW372975	MW373034
42	*V. pseudo-orobus* Fisch. & C.A.Mey.	*Cracca*	*Vicilla*	Choi et al., 2006	170901	Hapcheon, Gyeongnam, South Korea	N 35° 46′ 50.7″ E 128° 07′ 10.7″	KVA_RDA-2017-019	MW374778	MW372976	MW373035
43					170916	Cheongsong, Gyeongbuk, South Korea	N 36° 23′ 27.2″ E 129° 08′ 42.6″	KVA_RDA-2017-020	MW374779	MW372977	MW373036
44	*V. nipponica* Matsum.	*Cracca*	*Vicilla*	Choi et al., 2006	180811	Hongcheon, Gangwon, South Korea	N 37° 43′ 41.6″ E 128° 27′ 11.2″	KVA_RDA-2018-052	MW374780	MW372978	MW373037
45					170811	Hongcheon, Gangwon, South Korea	N 37° 42′ 50.3″ E 128° 26′ 49.3″	KVA_RDA-2017-021	MW374781	MW372979	MW373038
46					180810	Hoengseong, Gangwon, South Korea	N 37° 35′ 29.8″ E 128° 16′ 50.8″	KVA_RDA-2018-053	MW374782	MW372980	MW373039
47	*V. chosenensis* Ohwi	*Cracca*	*Vicilla*	Choi et al., 2006	170830	Pyengchang, Gangwon, South Korea	N 37° 39′ 59.59″ E 128° 39′ 22.25″	KVA_RDA-2017-022	MW374783	MW372981	MW373040
48					180831	Samchuk, Gangwon, South Korea	N 37° 13′ 40.42″ E 129° 7′ 24.3″	KVA_RDA-2018-054	MW374784	MW372982	MW373041
49					180926	Cheongsong, Gyeongbuk, South Korea	N 36° 26′ 32.25″ E 129° 8′ 24.3″	KVA_RDA-2018-055	MW374785	MW372983	MW373042
50	*V. bungei* Ohwi	*Cracca*	*Americanae*	Choi et al., 2006	180509	Samchuk, Gangwon, South Korea	N 37° 26′ 30.4″ E 129° 09′ 14.62″	KVA_RDA-2018-056	MW374786	MW372984	MW373043
51					170927	Gangneung, Gangwon, South Korea	N 37° 34′ 32″ E 128° 56′ 10″	KVA_RDA-2017-023	MW374787	MW372985	MW373044
52					180526	Gangneung, Gangwon, South Korea	N 37° 34′ 38.7″ E 128° 56′ 9.9″	KVA_RDA-2018-057	MW374788	MW37298	MW373045
53	*V. angustifolia* var. *segetilis* (Thuill.) K.Koch.	*Vicia*	*Vicia*	Leht, 2009	170503	Jeju, Jeju-do, South Korea	N 35° 49′ 53.9″ E 127° 4′ 36.04″	KVA_RDA-2017-024	MW374789	MW372987	MW373046
54					170508	Gerong, chungnam, South Korea	N 36° 18′ 45.68″ E 127° 14′ 0.21″	KVA_RDA-2017-025	MW374790	MW372988	MW373047
55	*V. hirsuta* (L.) Gray	*Cracca*	*Lenticula*	Jalilian et al., 2014	170503	Jeonju, Jeonbuk, South Korea	N 35° 49′ 53.9″ E 127° 4′ 36.04″	KVA_RDA-2017-026	MW374791	MW372989	MW373048
56					170513	Buan, Jeonbuk, South Korea	N 35° 40′ 52.2″ E 126° 32′ 20.73″	KVA_RDA-2017-027	MW374792	MW372990	MW373049
57					180509	Gangneung, Gangwon, South Korea	N 37° 34′ 38.7″ E 128° 56′ 9.9″	KVA_RDA-2018-058	MW374793	MW372991	MW373050
58	*Vicia tetrasperma* (L.) Schreb.	*Cracca*	*Ervum*	Kupicha, 1976; Leht, 2009	180509	Gangneung, Gangwon, South Korea	N 37° 34′ 38.7″ E 128° 56′ 9.9″	KVA_RDA-2018-059	MW374794	MW372992	MW373051
59					170503	Jeonju, Jeonbuk, South Korea	N 35° 49′ 53.9″ E 127° 4′ 36.04″	KVA_RDA-2017-028	MW374795	MW372993	MW373052

### Polymerase Chain Reaction Amplification and Sequencing

The sequences of the universal primers for ITS2, *matK*, *rbcl*, and general PCR reaction conditions were obtained from previous studies (Chen et al., 2010; Raveendar et al., 2015). Amplification reactions were carried out in a total volume of 20 μl containing 10× PCR buffer, 0.1 mM primers, 0.2 mM each deoxyribonucleotide triphosphate (dNTP), 1 U Taq DNA polymerase (Inclone, South Korea), and 200 ng template DNA. High-quality PCR products were sequenced from both directions by Macrogen, South Korea to reduce sequencing error. All sequences were submitted to the National Center for Biotechnology Information (NCBI; Table 1).

### Data Analysis and Phylogenetic Analysis Based on Barcoding Regions

Consensus sequences of barcoding regions (ITS2, *matK*, and *rbcL*) were manually edited using the BioEdit program (ver.7.0.9, Tom Hall Ibis Biosciences, United States). Sequences were aligned using the “ClustalW” package in MEGA version X (Kumar et al., 2018) and were evaluated for nucleotide variation, number of segregating sites, and informative sites. Nucleotide diversity (π) analysis and Tajima’s test (D) were conducted in DnaSP version 5.10.01 (Librado and Rozas, 2009). To assess individual-level discrimination rates for each single marker and all possible combinations, “Best Match” and “Best Close Match” analysis was carried out in the TAXONDNA software package (Meier et al., 2006). For phylogenetic analysis, maximum likelihood trees were obtained using IQ-TREE version 1.6.2 software (Nguyen et al., 2015). In IQ-TREE, the Best Fit Substitution Model option was selected based on the Bayesian information criterion. Model tests were conducted for each region, and Best Fit Substitution Models were obtained for ITS2 (K2P + I), *matK* (GTR + F), *rbcL* (JC + I), ITS2 + *matK* (TIM + G4), *matK* + *rbcL* (TVM + G4), ITS2 + *rbcL* (TNE + I), and *ITS2* + *matK* + *rbcL* (TIM + G). Maximum likelihood trees were tested with bootstrap analysis adjusted to 1000 nonparametric bootstrap replicates in which *Trifolium repens* L. was used as outgroup. Species discrimination was considered successful only when all conspecific individuals generated a monophyletic clade, and species identification was evaluated using bootstrap values (Liu et al., 2011).

### Phylogenetic Analysis Based on Morphological Data

A list of 39 *Vicia* morphological characteristics was compiled from previous studies (Kupicha, 1976; Choi et al., 2006; Jalilian et al., 2014). The morphological characteristics of plant specimens were studied using a stereo-microscope (Olympus SZ61, Olympus, Tokyo, Japan). A digital Vernier caliper (CD-15APX, Mitutoyo, Kawasaki, Japan) was used to measure quantitative characteristics. As mentioned, all 39 morphological characters were evaluated and scored for morphological phylogenetic analysis. The characters and character states are listed in Table 2, and the data matrix is given in Supplementary Table S2. Maximum parsimony analysis was carried out in TNT 1.5 (Goloboff and Catalano, 2016). Scores for 19 taxa were constructed, and inapplicable states were denoted as “–.” Characteristic optimization was performed in MRBAyES SLAVER 5.30 (Nixon, 2008), and cladograms of the useful characteristics were visualized using Mesquite 3.61 (Maddison and Maddison, 2019). The morphological characteristics of *V. hirsuta* was used as outgroup in the phylogenetic analysis.

**TABLE 2:** List of morphological characteristics.

No.	Character	Character states
1	Life form	0, annual or biannual; 1, perennial
2	Growth habit	0, climbing; 1, erect; 2, ascending
3	Tuber presence	0, absent; 1, present
4	Stem height	0, small (up to 40 cm); 1, high (over 40 cm)
5	Stem form	0, slender; 1, rigid
6	Stem pubescence	0, glabrous; 1, sparse (epidermis visible); 2, dense (epidermis not visible)
7	Leaflet pairs per leaf	0, 1 pair; 1, more than two pairs
8	Number of leaflets	0, 2; 1, 4–8; 2, 6–14; 3, 16–24
9	Leaflet size relative to leaf	0, same size; 1, larger at leaf base
10	Leaflet length	0, <20 mm; 1, 20–40 mm; 2, >40 mm
11	Leaflet width	0, <10 mm; 1, 10–20 mm; 2, >20 mm
12	Leaflet shape	0, elliptic; 1, lanceolate; 2, oblong; 3, obovate; 4, ovate
13	Leaflet apex	0, acuminate; 1, acute; 2, obtuse; 3, truncate
14	Leaflet base	0, cuneate; 1, obtuse
15	Leaflet adaxial hair density	0, glabrous; 1, sparse (epidermis visible); 2, dense (epidermis not visible)
16	Leaflet abaxial hair density	0, glabrous; 1, sparse (epidermis visible); 2, dense (epidermis not visible)
17	Stipule nectariferous spot	0, absent; 1, present
18	Stipule present	0, absent; 1, present
19	Stipule surface	0, smooth; 1, hairy
20	Tendrils	0, absent; 1, present; 2, on some leaves
21	Tendril branching	0, unbranched; 1, branched
22	Tendril hair density	0, glabrous; 1, sparse (epidermis visible); 2, dense (epidermis not visible)
23	Number of flowers per inflorescence	0, 1–(2); 1, 2–4; 2, 5, or more
24	Relative length of limb and claw in standard	0, shorter than claw 1, as long as; 2, longer than claw
25	Standard shape	0, oblong; 1, stenonychinoid; 2, platonychinoid
26	Standard color pattern	0, absent; 1, differently colored spot; 2, differently colored veins; 3, differently colored back; 4, darker
27	Standard color	0, white; 1, yellow; 2, purple or bluish
28	Wing color	0, white; 1, yellow; 2, purple (bluish)
29	Wing length	0, 1/4 shorter than the standard; 1, slightly shorter than standard; 2, longer than standard 3, similarity to standard
30	Calyx teeth length	0, equal; 1, unequal
31	Seed shape	0, spherical; 1, oblong
32	Seed color	0, brown; 1, greenish brown; 2, reddish brown; 3, greenish yellow
33	Seed color mottling	0, absent; 1, present
34	Hilum shape	0, circumlinear; 1, linear; 2, oblong; 3, wedge; 4, oval
35	Hilum color	0, pale; 1, same as seed color; 2, dark
36	Seed size	0, <3 mm; 1, 3–5(6) mm; 2, >6 mm
37	Style shape	0, terete; 1, dorsally compressed; 2, laterally compressed
38	Style pubescence	0, evenly pubescent; 1, tufted abaxially; 2, v-shaped; 3, tufted adaxially
39	Ovary hairiness	0, glabrous; 1, glabrous hairy; 2, short glandular hairy

## Results

### Characteristics and Genetic Distances of Barcoding Regions

As a result of amplification with universal primers (ITS2, *matK*, and *rbcL*) using template gDNA extracted from each sample, the sequence length of ITS2 varied from 339 to 355 bp, whereas *matK* and *rbcL* measured 705 and 542 bp, respectively (Table 3). Most *Vicia* species had amplified ITS2 sequences of 352 bp in length, whereas *Vicia dasycarpa* Ten. had sequences of 342 bp, *V. villosa* and *V. cracca* of 339 bp, and *V. hirsuta* and *V. tetrasperma* of 355 bp. The ratio of G to C was high in the ITS2 region (48–51%) and low in the *matK* region (30–32%). In the aligned dataset, the lengths of ITS2, *matK*, and *rbcL* were 358, 705, and 542 bp, respectively. Deletion sites were found at 64–76 bp in *V. dasycarpa*, *V. villosa*, and *V. cracca*, whereas insertion sites were found at 81–83 bp in *V. dasycarpa* and at 214–216 bp in *V. hirsuta* and *V. tetrasperma*. Nucleotide diversity (π) was analyzed for each locus and was calculated as 0.02109 for *matK*, 0.01262 for ITS2, and 0.00819 for *rbcL*. When comparing the nucleotide diversity (π) of the barcode combinations, the ITS2 + *matK* combination produced the highest value, at 0.01837, followed by *matK* + *rbcL*, ITS2 + *matK* + *rbcL*, and ITS2 + *rbcL* at 0.01551, 0.01489, and 0.00990, respectively. *matK* possessed the largest mean intraspecific distance, at 0.00126, followed by ITS2 + *matK*, *matK* + *rbcL*, ITS2, ITS2 + *matK* + *rbcL*, ITS2 + *rbcL*, and *rbcL* at 0.00101, 0.00083, 0.00075, 0.00075, 0.00048, and 0.00031, respectively. Similarly, ITS2 possessed the largest mean interspecific distance, at 0.028, followed by ITS2 + *matK*, matK, ITS2 + *matK* + *rbcL*, *matK* + *rbcL*, ITS2 + *rbcL*, and *rbcL* at 0.0248, 0.0231, 0.0195, 0.0171, 0.0167, and 0.0093, respectively (Table 4).

**TABLE 3:** Characteristics and variation of the ITS2, *matK*, and *rbcL* region.

Taxon	ITS2				*matK*				*rbcL*			
	Length (bp)	G + C (%)	Intraspecific distance	Interspecific distance	Length (bp)	G + C (%)	Intraspecific distance	Interspecific distance	Length (bp)	G + C (%)	Intraspecific distance	Interspecific distance
*V. dasycarpa*	342	50	0.000	0.018	705	31	0.000	0.035	542	42	0.000	0.013
*V. villosa*	339	49	0.000	0.023	705	30	0.000	0.029	542	43	0.000	0.012
*V. cracca*	339	48	0.000	0.024	705	31	0.000	0.032	542	42	0.000	0.017
*V. amoena*	352	50	0.000	0.009	705	32	0.000	0.019	542	43	0.000	0.005
*V. amurensis*	352	49	0.000	0.012	705	32	0.001	0.022	542	43	0.001	0.006
*V. japonica*	352	50	0.004	0.013	705	32	0.005	0.017	542	43	0.000	0.005
*V. unijuga* var. *kaussanensis*	352	50	0.000	0.011	705	32	0.000	0.015	542	43	0.000	0.005
*V. unijuga*	352	50	0.000	0.011	705	32	0.000	0.015	542	43	0.000	0.005
*V. linearifolia*	352	50	0.000	0.011	705	32	0.000	0.015	542	43	0.000	0.005
*V. unijuga* f. *angustifolia*	352	50	0.000	0.011	705	32	0.000	0.015	542	43	0.000	0.005
*V. hirticalycina*	352	50	0.000	0.012	705	32	0.000	0.015	542	43	0.000	0.005
*V. venosa* var. *cuspidata*	352	50	0.008	0.011	705	32	0.005	0.016	542	42-43	0.001	0.006
*V. pseudo-orobus*	352	50	0.000	0.011	705	32	0.003	0.015	542	42	0.000	0.007
*V. nipponica*	352	50	0.000	0.011	705	32	0.000	0.015	542	43	0.000	0.005
*V. chosenensis*	352	50	0.000	0.009	705	31	0.000	0.018	542	43	0.000	0.005
*V. bungei*	352	51	0.000	0.015	705	31	0.001	0.028	542	42	0.000	0.009
*V. angustifolia* var. *segetilis*	352	49	0.000	0.028	705	31	0.000	0.040	542	43	0.000	0.016
*V. hirsuta*	355	50	0.000	0.025	705	30	0.002	0.039	542	43	0.000	0.028
*V. tetrasperma*	355	50	0.000	0.018	705	30	0.003	0.034	542	43	0.000	0.012

**TABLE 4:** Genetic diversity of barcoding markers used in this study.

DNA barcode	Individuals (*n*)	Aligned length	Number of segregating sites	Nucleotide diversity (π)	Tajima’s test (*D*)	Mean intraspecific distance	Mean interspecific distance	Interspecific/intraspecific distance ratio	Species resolved (%)	Discrimination (%)
ITS2	59	358	26	0.01262	−1.09646	0.00075	0.0280	37.376	94.9	47.4
*matK*	59	705	78	0.02109	−0.43438	0.00126	0.0231	18.368	98.1	52.6
*rbcL*	59	542	31	0.00819	−1.09090	0.00031	0.0093	30.483	91.4	36.8
ITS2 + *matK*	59	1063	105	0.01837	−0.66370	0.00101	0.0248	24.509	98.7	57.9
ITS2 + *rbcL*	59	900	57	0.00990	−1.14721	0.00048	0.0167	34.451	95.0	47.4
*matK* + *rbcL*	59	1247	110	0.01551	−0.66587	0.00083	0.0171	20.674	97.0	52.6
ITS2 + *matK* + *rbcL*	59	1605	136	0.01489	−0.77983	0.00075	0.0195	25.912	99.1	57.9

### Intraspecific and Interspecific Variability in *Vicia* Species

The intraspecific and interspecific variability of each barcode region was analyzed in 19 *Vicia* species. The results showed that there was no intraspecific variability among the barcode regions in 12 of the 19 taxa. The intraspecific variability of ITS2 was 0.000–0.008, and *Vicia japonica* A. Gray and *Vicia venosa* var. *cuspidata* Maxim. measured 0.004 and 0.008, respectively. The intraspecific variability of *matK* was 0.000–0.005, with *V. japonica* and *V. venosa* var. *cuspidata* each measuring 0.005, *V. pseudo-orobus* and *V. tetrasperma* each measuring 0.003, *V. hirsuta* measuring 0.002, and *Vicia bungei* Ohwi and *Vicia amurensis* Oett. each measuring 0.001. The intraspecific variability of *rbcL* was 0.000–0.001, with *V. amurensis* and *V. venosa* var. *cuspidata* each measuring 0.001. Interspecific variability was discovered in all species, with ITS2 measuring in the range of 0.009–0.028, *matK* in the range of 0.015–0.040, and *rbcL* in the range of 0.005–0.028. *V. angustifolia* var. *segetilis* K. Koch. produced the highest interspecific variability values for ITS2 and *matK* of 0.028 and 0.040, respectively, and *V. hirsuta* possessed the highest interspecific variability in *rbcL* of 0.028.

### Sequence Similarity for Efficient Species Discrimination

TAXONDNA analysis using the “Best Match” and “Best Close Match” methods was used to evaluate the species discrimination capabilities of each barcode region. The results showed similar discrimination successes among regions (Table 5). Of the three single-locus barcodes, ITS2 and *matK* showed the highest success rates (54.23%) for correct identification of species, and *rbcL* showed a success rate of 38.98%. Success rate (59.32%) was higher among four combination barcodes, and the success rates of the ITS2 + *matK* + *rbcL* and ITS2 + *matK* combinations were similar.

**TABLE 5:** Number (rates) of sample identification based on the analysis of the “best match” and “best close match” functions using TAXONDNA software for each DNA barcoding marker and combinations from 59 individuals.

Barcoding regions	Best match, *N* (%)			Best close match, *N* (%)			
	Correct	Ambiguous	Incorrect	Correct	Ambiguous	Incorrect	No match
ITS2	32 (54.23)	27 (45.76)	0 (0.0)	32 (54.23)	27 (45.76)	0 (0.0)	0 (0.0)
*matK*	32 (54.23)	26 (44.06)	1 (1.69)	32 (54.23)	26 (44.06)	1 (1.69)	0 (0.0)
*rbcL*	23 (38.98)	35 (59.32)	1 (1.69)	23 (38.98)	35 (59.32)	1 (1.69)	0 (0.0)
ITS2 + *matK*	35 (59.32)	23 (38.98)	1 (1.69)	35 (59.32)	23 (38.98)	1 (1.69)	0 (0.0)
ITS2 + *rbcL*	32 (54.23)	26 (44.06)	1 (1.69)	32 (54.23)	26 (44.06)	1 (1.69)	0 (0.0)
*matK* + *rbcL*	32 (54.23)	24 (40.67)	3 (5.08)	32 (54.23)	24 (40.67)	3 (5.08)	0 (0.0)
ITS2 + *matK* + *rbcL*	35 (59.32)	21 (35.59)	3 (5.08)	35 (59.32)	21 (35.59)	3 (5.08)	0 (0.0)

### Phylogenetic Analysis and Species Identification

**FIGURE 2:**
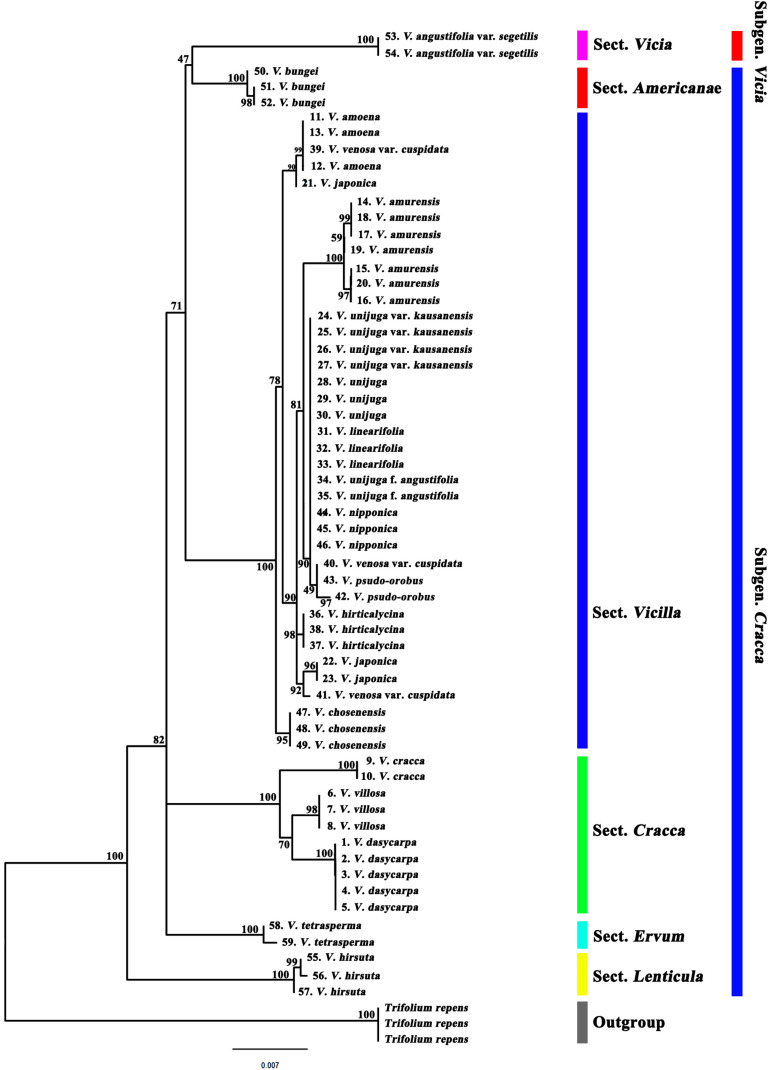
Phylogenetic analysis of 19 *Vicia* species based on the nucleotide sequences of the combined ITS2 + *matK* + *rbcL* regions.

**FIGURE 3:**
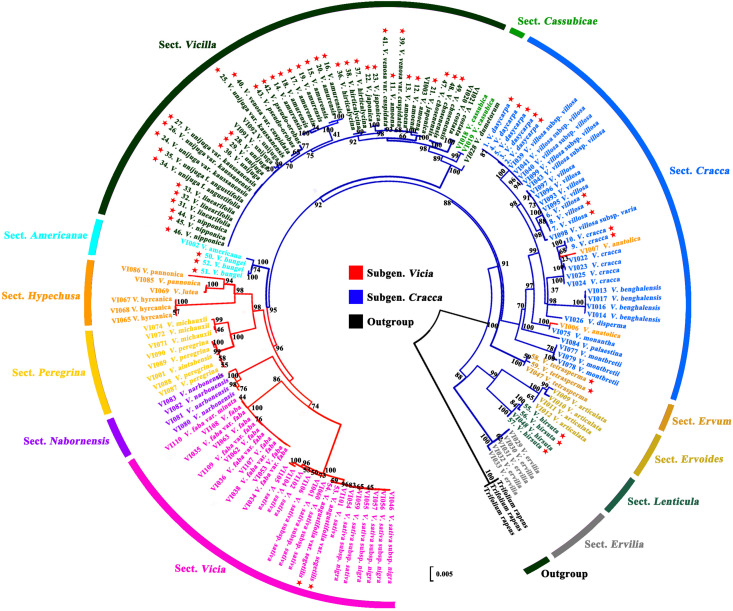
Phylogenetic analysis of *Vicia* species based on the nucleotide sequences the combined ITS2 + *matK* + *rbcL* regions. ★ Collected accessions of native *Vicia* species in this study.

Phylogenetic analysis of 19 Korean *Vicia* taxa (59 accessions) was performed using the sequences of single regions (ITS2, *matK*, and *rbcL*) and of combinations of regions (ITS2 + *matK*, ITS2 + *rbcL*, *matK* + *rbcL*, and ITS2 + *matK* + *rbcL*). The *Trifolieae* tribe is known to be a sister of the monophyletic *Fabeae* tribe and was used as an outgroup. Maximum likelihood trees showed that the *Vicia* species were clearly monophyletic, as each section showed clear discrimination (Figures 2, 3 and Supplementary Figures S1–6). It was confirmed that the ITS2 region separated the species into two clades (clades 1 and 2), and species of sect. *Vicilla* were grouped into clade 1. Clade 2 was divided into two subclades, with *V. angustifolia* var. *segetilis* and *V. bungei* in subclade 1 and species of sect. *Cracca*, *V. hirsuta*, and *V. tetrasperma* in subclade 2. Based on the phylogenetic tree, the species resolution and discrimination rate of ITS2 were 94.9 and 47.4%, respectively (Supplementary Figure S1 and Table 4). The *matK* region also separated the species into two clades (clades 1 and 2), and *V. hirsuta* was grouped into clade 1. Clade 2 was divided into three subclades, with *V. angustifolia* var. *segetilis*, *V. bungei*, and species of sect. *Vicilla* grouped into subclade 1, species of sect. *Cracca* clustered into subclade 2, and *V. tetrasperma* in subclade 3. The species resolution and discrimination rate of *matK* were 98.1 and 52.6%, respectively (Supplementary Figure S2 and Table 4). Similarly, the *rbcL* region also separated the species into two clades (clades 1 and 2), with *V. hirsuta* found in clade 1. Clade 2 was divided into three subclades, with *V. tetrasperma* grouped into subclade 1, *V. angustifolia* var. *segetilis* and species of sect. *Vicilla* found in subclade 2, and species of sect. *Cracca* being grouped into subclade 3. Based on the phylogenetic tree, the species resolution and discrimination rate of *rbcL* were 91.4 and 36.8%, respectively (Supplementary Figure S3 and Table 4). Combining loci improved species discrimination, as the ITS2 + *matK* + *rbcL* and ITS2 + *matK* combinations each produced a value of 57.9%, and ITS2 + *rbcL* and *matK* + *rbcL* produced values of 47.4 and 52.6%, respectively (Supplementary Figures S4–6 and Table 4). The ITS2 + *matK* + *rbcL* combination provided the highest species resolution (99.1%), and the *rbcL* locus alone produced the lowest resolution, at 91.4%.

Barcode sequence of ITS2, *matK*, and *rbcL* were downloaded from the NCBI and combined with the present study. The results showed that each section of *Vicia* appeared to be monophyletic from the outgroup and formed six groups (Figure 3). Sect. *Ervilia*, sect. *Lenticula*, and sect. *Ervoides* are clustered in group 1; sect. *Ervum* is clustered in group 2; sect. *Cracca* is clustered in group 3; *Cassubicae* and sect. *Vicilla* clustered together in group 4; sect. *Americanae* found in group 5; and sect. *Hypechusa*, sect. *Peregrina*, sect. *Narbonensis*, and sect. *Vicia* is clustered into group 6. Almost all Korean species of *Vicia*, excluding *V. angustifolia* var. *segetilis* (subgenus *Vicia*), included the subgenus *Cracca.* Most Korean *Vicia* species, such as *Vicia amoena* Fisch. ex DC., *V. amurensis*, *V. japonica*, *V. unijuga* var. *kaussanensis*, *V. unijuga*, *V. linearifolia*, *V. unijuga* f. *angustifolia* Makino ex Ohwi, *V. hirticalycina*, *V. venosa* var. *cuspidata*, *V. pseudo-orobus*, *V. nipponica*, and *V. chosenensis* are clustered in sect. *Vicilla* (group 4), and three *Vicia* species (*V. dasycarpa*, *V. villosa*, and *V. cracca*) are grouped into sect. *Cracca* (group 3). *V. bungei*, *V. angustifolia* var. *segetilis*, *V. hirsuta*, and *V. tetrasperma* are clustered into sect. *Americanae* (group 5), sect. *Vicia* (group 6), sect. *Lenticula* (group 1), and sect. *Ervum* (group 2), respectively.

### Morphological Analysis of Species Identification

**FIGURE 4:**
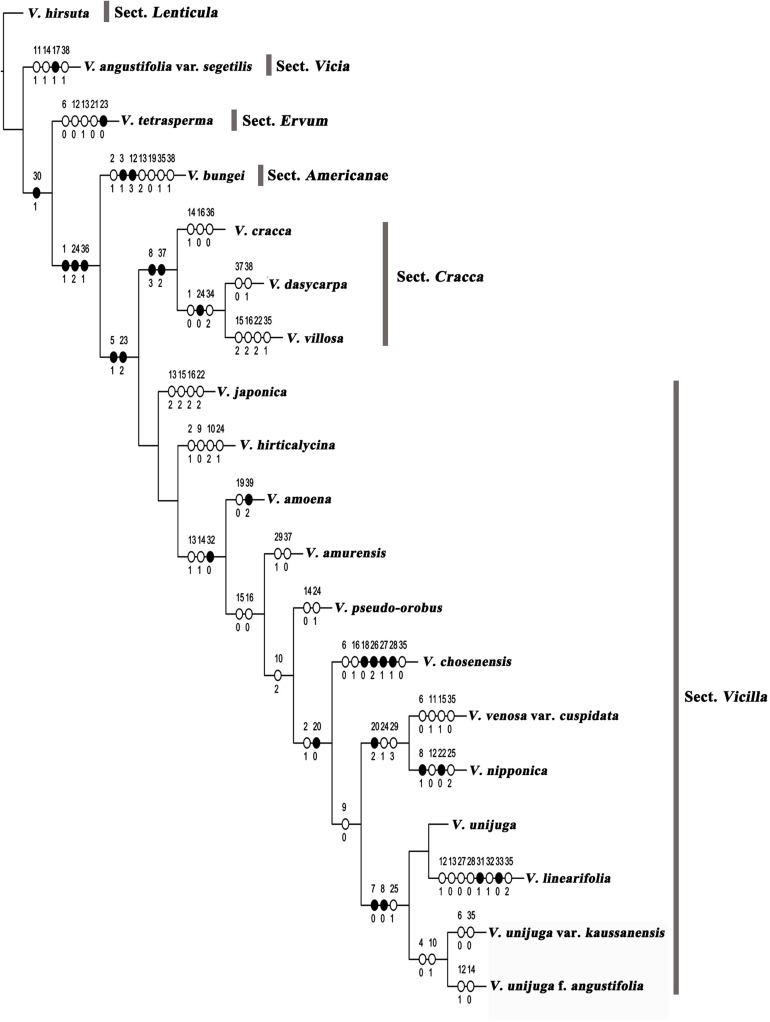
Character support for phylogenetic positions of *Vicia* species. Strict consensus tree (length = 135 steps; CI = 0.48; RI = 0.56) showing the character state optimizations at each node of the cladogram, represented by circles. Non-homoplastic changes in character states are represented by black circles, and homoplastic changes are shown with white circles. The numbers above and below each circle represent the character and character state number, respectively. Matrix information on characters and character state numbers: (1) life form (0, annual or biannual; 1, perennial); (2) growth habit (0, climbing; 1, erect; 2, ascending); (3) tuber presence (0, absent; 1, present); (4) stem height [0, small (up to 40 cm); 1, high (over 40 cm)]; (5) stem form (0, slender; 1, rigid); (6) stem pubescence [0, glabrous; 1, sparse (epidermis visible); 2, dense (epidermis not visible)]; (7) leaflet pairs per leaf (0, one pair; 1, more than two pairs); (8) number of leaflets (0, 2; 1, 4–8; 2, 6–14; 3, 16–24); (9) leaflet size relative to leaf (0, same size; 1, larger at leaf base); 10, leaflet length (0, <2.0 mm; 1, 20–40 mm; 2, >40 mm); (11) leaflet width (0, <10 mm; 1, 10–20 mm; 2, >20 mm); (12) leaflet shape (0, elliptic; 1, lanceolate; 2, oblong; 3, obovate; 4, ovate); (13) leaflet apex (0, acuminate; 1, acute; 2, obtuse; 3, truncate); (14) leaflet base (0, cuneate; 1, obtuse); (15) leaflet adaxial hair density [0, glabrous; 1, sparse (epidermis visible); 2, dense (epidermis not visible)]; (16) leaflet abaxial hair density [0, glabrous; 1, sparse (epidermis visible); 2, dense (epidermis not visible)]; (17) stipule nectariferous spot (0, absent; 1, present); (18) stipules present (0, absent; 1, present); (19) stipule surface (0, smooth; 1, hairy); (20) tendrils (0, absent; 1, present; 2, on some leaves); (21) tendril branching (0, unbranched; 1, branched); (22) tendril hair density [0, glabrous; 1, sparse (epidermis visible); 2, dense (epidermis not visible)]; (23) number of flowers per inflorescence [0, 1–(2); 1, 2–4; 2, 5 or more]; (24) relative length of limb and claw in standard (0, shorter than claw; 1, as long as; 2, longer than claw); (25) standard shape (0, oblong; 1, stenonychinoid; 2, platonychinoid); (26) standard color pattern (0, absent; 1, differently colored spot; 2, differently colored veins; 3, differently colored back; 4, darker); (27) standard color (0, white; 1, yellow; 2, purple or bluish); (28) wing color [0, white; 1, yellow; 2, purple (bluish)]; (29) wing length (0, 1/4 shorter than the standard; 1, slightly shorter than standard; 2, longer than standard; 3, similar to standard); (30) calyx teeth length (0, equal; 1, unequal); (31) seed shape (0, spherical; 1, oblong); (32) seed color (0, brown; 1, greenish brown; 2, reddish brown; 3, greenish yellow); (33) seed color mottling (0, absent; 1, present); (34) hilum shape (0, circumlinear; 1, linear; 2, oblong; 3, wedge; 4, oval); (35) hilum color (0, pale; 1, seed color; 2, dark); (36) seed size (0, <3 mm; 1, 3–5(6) mm; 2, >6 mm); (37) style shape (0, terete; 1, dorsally compressed; 2, laterally compressed); (38) style pubescence (0, evenly pubescent; 1, abaxially tufted; 2, v-shaped; 3, adaxially tufted); (39) ovary hairiness (0, glabrous; 1, glabrous hairy; 2, short glandular hairy).

**FIGURE 5:**
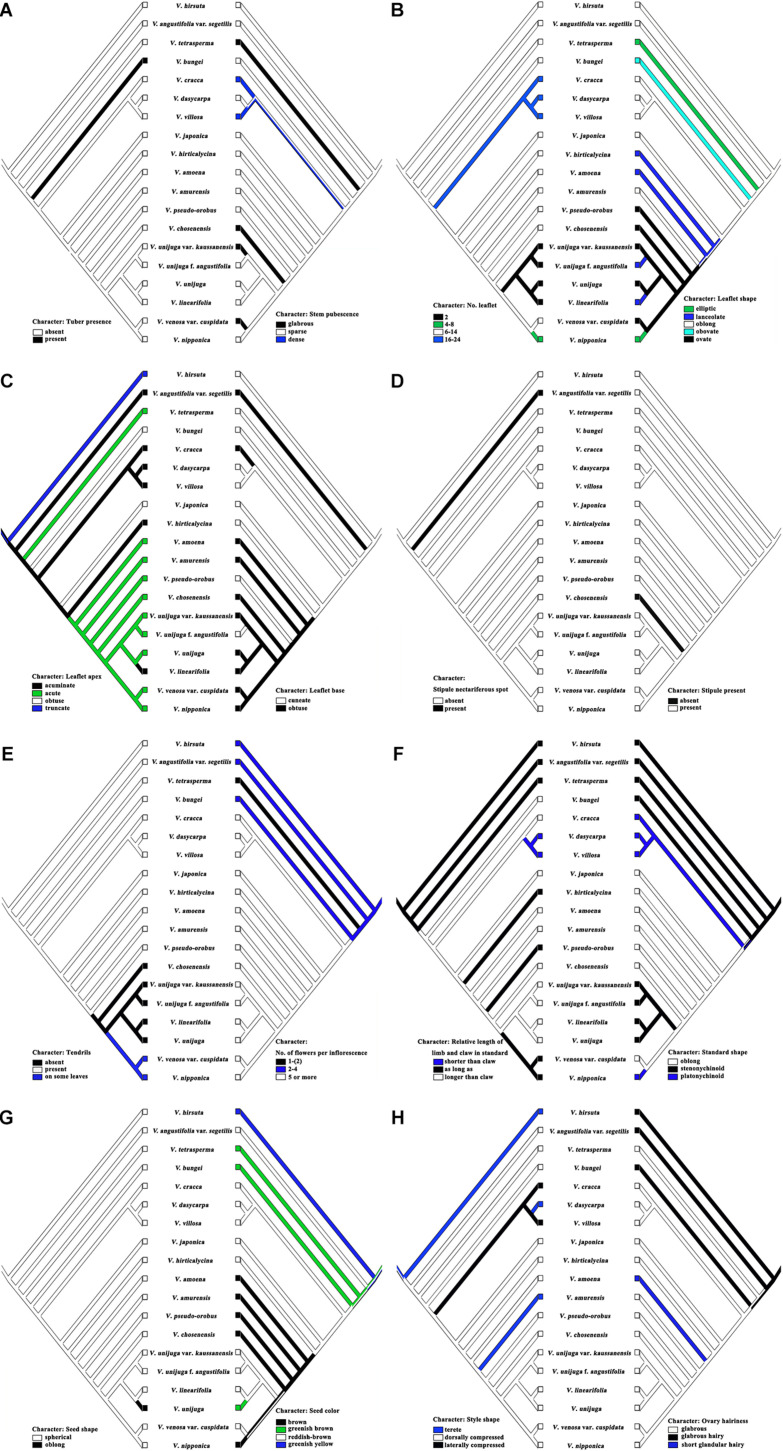
Character state mapping of 16 useful morphological characteristics. **(A)** Character of Tuber presence and Stem pubescence; **(B)** Character of No. of leaflets and Leaflet shape; **(C)** Character of Leaflet apex and Leaflet base; **(D)** Character of Stipule nectariferous spot and Stipule present; **(E)** Character of Tendrils and No. of flowers per inflorescence; **(F)** Character of relative length of limb and claw in standard and Standard shape; **(G)** Character of Seed shape and Seed color; **(H)** Character of Style shape and Ovary hairiness.

Maximum parsimony analysis was performed to identify the optimal morphological traits for efficient identification of Korean *Vicia* species (Figures 4, 5). The results of character support for phylogenetic positions and character state mapping and 16 useful morphological characteristics, such as tuber presence, stem pubescence, number of leaflet, leaflet shape, leaflet apex, leaflet base, stipule nectariferous spot, stipule present, tendrils, number of flower per inflorescence, relative length of limb and claw in standard, standard shape, seed shape, seed color, style shape, and ovary hairiness were selected for *Vicia* species identification (Figure 5). *V. hirsuta* was used as the outgroup as it is a first *Vicia* species found to diverge from species of the *Trifolieae* tribe, a known sister to the monophyletic *Fabeae*, of *Vicia ervilia* and *Vicia sylvatica*. *V. hirsuta* has white flowers and greenish yellow seed color, and small-sized seeds separate the species from others in the *Vicia* genus. Cladistic analysis revealed that *V. angustifolia* var. *segetilis* of sect. *Vicia* was separated from *V. hirsuta* by the following characteristics: leaflet width of 10–20 mm, obtuse leaflet base, present stipule nectariferous spot, and abaxially tufted style pubescence and that the other species of sections were supported by unequal calyx teeth length. Characteristic of present nectaferious spot can be a useful trait to distinguish *V. angustifolia* var. *segetilis* (Figure 5D). *V. tetrasperma* of sect. *Ervum* was separated by the following characteristics: glabrous stem pubescence, elliptic leaflet shape, acute leaflet apex, unbranched tendrils, and 1–(2) flower per inflorescence and that the other species of sections were supported by characteristics of life form, relative length of limb and claw in standard, and seed size. Characteristic of 1–(2) flower per inflorescence can be a useful trait to distinguish *V. tetrasperma* (Figure 5E). *V. bungei* is separated by its being erect, the presence of tubers, obovate leaflet shape, obtuse leaflet apex, smooth stipule surface, hilum color that is same as the seed color, and abaxially tufted style pubescence. Characteristic of the presence of tubers can be a useful trait to distinguish *V. bungei* (Figure 5A).

Species from sect. *Cracca* and sect. *Vicilla* formed a sister clade because of their rigid stems and the fact that they form five or more flowers per inflorescence. Species of sect. *Cracca* were supported by their characteristic of 16–24 leaflets. *V. cracca* was separated by its obtuse leaflet base, glabrous leaflet abaxial hair density, and the fact that its seeds are <3 mm in size. Characteristics of 16–24 leaflets and obtuse leaflet base can be useful traits to distinguish *V. cracca* (Figures 5B,C). *V. dasycarpa* and *V. villosa* were supported by their annual or biannual life form, limb of standard that is shorter than the claw, and their oblong hilum shape. *V. dasycarpa* was distinguished by its terete style shape and abaxially tufted style pubescence, and *V. villosa* was distinguished owing to its dense leaflet adaxial hair density, dense leaflet abaxial hair density, dense tendril hair density, and hilum color that is same as the seed color. Characteristics of a limb that is shorter than the claw and terete style shape can be useful traits to distinguish *V. dasycarpa* (Figures 5F,H), and characteristics of limb a that is shorter than the claw and laterally compressed style shape can be useful traits to distinguish *V. villosa* (Figures 5F,H). *V. japonica* was separated by its obtuse leaflet apex, dense leaflet adaxial hair density, dense leaflet abaxial hair density, and dense tendril hair density. Characteristics of oblong standard shape and obtuse leaflet apex can be useful traits to distinguish *V. japonica* (Figures 5C,F). *V. hirticalycina* was separated by its erect, same size leaflet, leaflet length that is more than 40 mm, and limb that is as long as the claw. Characteristics of five or more flower per inflorescence and acuminate leaflet apex can be useful traits to distinguish *V. hirticalycina* (Figures 5C,E). *V. amoena* Fisch. ex DC. was separated by its smooth surface and short glandular hairy ovaries. Characteristic of short glandular hair of the ovary can be a useful trait to distinguish *V. amoena* (Figure 5H). *V. amurensis* was separated by its wing that is slightly shorter than the standard and terete style shape. Characteristics of brown seed color and terete style shape can be useful traits to distinguish *V. amurensis* (Figures 5G,H). *V. pseudo-orobus* was separated by its cuneate leaflet base and limb that is as long as the claw. Characteristics of acute leaflet apex, present tendril, and limb that is as long as the claw can be useful traits to distinguish *V. pseudo-orobus* (Figures 5C,E,F). *V. chosenensis* was separated by its absent tendrils, glabrous stem pubescence, sparse leaflet abaxial hair density, absent stipule, standard differently colored veins, yellow standard color, yellow wing color, and pale hilum color. Characteristic of absent stipule can be a useful trait to distinguish *V. chosenensis* (Figure 5D).

The positions of *V. venosa* var. *cuspidata* and *V. nipponica* sister to *V. unijuga* A. Braun, *V. linearifolia* Y.N.Lee, *V. unijuga* var. *kaussanensis*, and *V. unijuga* f. *angustifolia* Makino were supported by the fact that their leaflets were all the same sizes. *V. venosa* var. *cuspidata* was differentiated by glabrous stem pubescence, 10–20 mm leaflet width, sparse leaflet adaxial hair density, and pale hilum color, and *V. nipponica* was distinguished because of having four to eight leaflets, elliptic leaflet shape, glabrous tendril hair density, and platonychinoid standard shape. Characteristics of the tendril of one some leaves and glabrous stem pubescence can be useful traits to distinguish *V. venosa* var. *cuspidata* (Figures 5A,E), and characteristics of the tendril of one leaf and four to eight leaflets can be useful a trait to distinguish *V. nipponica* (Figures 5B,E). *V. unijuga* was separated by the fact that it had one pair of leaflets, two leaflets in one pair of leaflets, and stenonychinoid standard shape. *V. linearifolia* was distinguished by its lanceolate leaflet shape, acuminate leaflet apex, white flower, oblong seed shape, greenish brown seed color, absent seed color mottling, and dark hilum color. Characteristics of two leaflets, sparse stem pubescence, and ovate leaflet shape can be useful traits to distinguish *V. unijuga* (Figures 5A,B). Characteristics of oblong seed shape and greenish brown seed color can be useful traits to distinguish *V. linearifolia* (Figure 5G). *V. unijuga* var. *kaussanensis* and *V. unijuga* f. *angustifolia* were supported by their characteristic of small stem height and 20–40 mm leaflet length. *V. unijuga* var. *kaussanensis* was separated by its glabrous stem pubescence and pale hilum color. *V. unijuga* f. *angustifolia* was separated by its lanceolate leaflet shape and cuneate leaflet base. Characteristics of two leaflets and glabrous stem pubescence can be useful traits to distinguish *V. unijuga* var. *kaussanensis* (Figures 5A,B), and characteristics of two leaflets and cuneate leaflet base can be useful traits to distinguish *V. unijuga* f. *angustifolia* (Figures 5B,C).

## Discussion

### Characteristics of Barcoding Regions in *Vicia*

The present study investigated the possibility of classifying and examining the molecular relationships among Korean *Vicia* species using three barcode regions (ITS2, *matK*, and *rbcL*). PCR amplification and sequencing of all three barcoding regions showed 100% success rate with Korean *Vicia* species. The universality of the PCR amplification success is an important criterion for DNA barcoding (Kress and Erickson, 2007). Barcode sequence alignment analysis revealed considerable nucleotide diversity (π) between the loci, in which ITS2 showed the largest mean interspecific distance (Table 4), whereas there was no intraspecific variability among the barcode regions in 12 of the 19 taxa. In general, plastid and rDNA regions were reported as potential loci for barcoding due to the low sequence divergence (Saski et al., 2007; Erickson et al., 2008). However, an ideal DNA barcode should be universal, reliable, cost effective, and provides considerable discriminatory power for plant species identification.

The present study showed, as single barcodes, that the species discrimination abilities of all three barcodes were poor (Supplementary Figures S1, 2), whereas the barcode combinations (ITS2 + *matK* + *rbcL*) improved species discrimination as well as species resolution (Table 4) as reported in the previous study (Raveendar et al., 2017). Similarly, in an earlier study, Wu et al. (2020) evaluated a combination of barcodes such as *matK*, *rbcL*, *trnH-psbA*, *trnL-trnF*, ITS1, and ITS2 to identify 161 *Vicia* species. However, the present study did not include all known *Vicia* species, as reported in previous studies (Choi et al., 2006; Raveendar et al., 2017; Wu et al., 2020). There are known endemic *Vicia* species in Korea, which have a restricted distribution that needed efficient conservation strategy. Moreover, collection and conservation of many *Vicia* species were available at the Korean gene bank. Hence, the present study was focused mainly on *Vicia* species distributed in South Korea together with available germplasm resources to preserve the biological diversity.

### Phylogenetic Analysis of Barcode Regions

The phylogenetic analysis of ITS2, *matK*, and *rbcL* were displayed sufficient markers for *V. angustifolia* var. *segetilis*, *V. bungei*, *V. villosa*, *V. cracca*, *V. dasycarpa*, *V. hirsuta*, *V. tetrasperma*, *V. amurensis*, *V. hirticalycina* Nakai, and *V. chosenensis* species identification, whereas *V. unijuga*, *V. unijuga* var. *kaussanensis*, *V. linearifolia*, *V. unijuga* f. *angustifolia*, and *V. nipponica* species did not form distinct clusters in the phylogenetic tree. In the phylogenetic analysis, *V. amoena*, *V. japonica*, *V. venosa* var. *cuspidata*, and *V. pseudo-orobus* were not differentiated, and these species were found to be very closely related to each other, which comes under sect. *Vicilla*. Phylogenetic analysis can help us to understand the evolutionary history of genes and species. Most importantly, the mode and time of species evolution can be estimated based on characteristics (Som, 2015). Phylogenetic analyses have previously been performed using morphological, anatomical, molecular, and karyological characteristics of the *Vicia* genus (Leht and Jaaska, 2002; Van de Wouw et al., 2003; Leht, 2005, 2009; Choi et al., 2006; Castiglione et al., 2011). The genus *Vicia* L. is included in the *Fabeae* (syn. *Vicieae*) tribe, along with four other genera: *Lathyrus* L., *Lens* Mill., *Pisum* L., and *Vavilovia* Fed. In previous studies, it was also reported that *Lathyrus*, *Lens*, *Pisum*, and *Vavilovia* are nested within *Vicia* species of all genera analyzed (Smýkal et al., 2011; Schaefer et al., 2012; Omar et al., 2019).

It is well known that most taxa in sect. *Vicilla* are Asian species distributed across Siberia, China, Mongolia, Japan, and South South Korea, with the exception of European taxa such as *Vicia dumetorum* and *V. sylvatica* (Li and Liu, 1996). Most of the native *Vicia* taxa in South Korea belong to sect. *Vicilla* (Figure 3), with Korean endemic species such as *V. chosenensis*, *V. hirticalycina*, and *V. unijuga* var. *kaussanensis* (Chung et al., 2017). Endo et al. (1996) defined the sect. *Amurense* Y.Endo & H.Ohashi to contain approximately four species: *V. amurensis*, *Vicia dichroantha* Diels, *Vicia nummularia* Hand.-Mazz., and *Vicia tibetica* Fisch. with similar morphological characteristics such as terete style, globose stigma, and swollen-type pollen mesocolpium. However, terete style was found to be polyphyletic; hence, sect. *Amurense* has not been reported as monophyletic, even in molecular phylogenetic studies (Endo et al., 2010). Schaefer et al. (2012) suggested that sect. *Vicilla* should be recircumscribed to include sect. *Amurense* and sect. *Cassubicae.* In the present study, *V. amurensis* was grouped in sect. *Vicilla* (Schur) Aschers & Graebner (Figure 3), thus supporting the infrageneric position of *V. amurensis* in sect. *Vicilla* (Kupicha, 1976; Rong and Shiyuan, 1994).

Species *V. bungei* distributed in China and Korea and *Vicia americana* Willd. distributed in North America are phenotypically and ecologically similar to each other, which is found in sect. *Americanae* (Kupicha, 1976; Endo et al., 2000). However, the two species can be discriminated by their differing root types and chromosome numbers. *V. bungei* has a well-developed root tuber and a chromosome number of 2*n* = 42 (hexaploid), whereas *V. americana* has no root tuber and has a chromosome number of 2*n* = 14 (diploid). Schaefer et al. (2012) suggested that the *V. americana* lineage may have spread from North America (*V. americana*) to Asia (*V. bungei*). Considering that the Bering land bridge connected northeastern Asia and northwestern North America in the Miocene, migration of flora over the land bridge could have occurred (Wen, 1999). Thus, *V. bungei* (hexaploid) may have evolved from the *V. americana* (diploid) lineage and could have developed tuber roots to adapt to cold stress or for energy storage purposes. In the current study, species of sect. *Americanae* formed sister clade with species of sections in the *Vicia* subgenus and were more closely related to sections in subgenus *Vicia* than sections in subgenus *Cracca.* In phylogenetic relationships analysis based on chloroplast protein-coding genes with 21 Papilionoideae subfamily, *V. bungei* was found to be closely related with the species of *Vicia faba*, *Vicia sativa*, and *Vicia sepium* in the *Vicia* subgenus (Yi et al., 2020). Therefore, the species of sect. *Americanae* may have played a key role in the evolution of subgenera *Vicia* and *Cracca.* More evolutionary study including geographic distribution is needed to confirm the evolutionary relationship of these species.

### Phylogenetic Analysis of Morphological Characteristics

The phylogenetic analysis based on 39 morphological characters well-supported the classification of species and sections of the genus *Vicia* in South Korea. The analysis revealed that tuber presence, stem pubescence, number of leaflets, leaflet shape, leaflet apex, leaflet base, stipule nectariferous spot, stipule present, tendrils, number of flowers per inflorescence, relative length of limb and claw in standard, standard shape, seed shape, seed color, style shape, and ovary hairiness were useful traits for section and species level discrimination (Figures 4, 5). Kupicha (1976) divided the genus *Vicia* into subgenus *Vicia* and subgenus *Cracca* by the presence or absence of stipule nectaries, which is known as a useful trait at the subgenus level. In the present study, of the *Vicia* species distributed in South Korea, only *V. angustifolia* var. *segetilis* had stipule nectaries (Figure 5). *V. bungei* of sect. *Americanae* was the only species with tubers, and this characteristic clearly distinguished *V. bungei* from other *Vicia* species (Endo et al., 2000). The number, length, and width of leaflets are useful characteristics for the primary classification of specific taxa in the genus *Vicia* (Fu et al., 1996; Abozeid et al., 2017). *V. cracca*, *V. villosa*, and *V. dasycarpa* of sect. *Cracca* possess 16–24 leaflets, and *V. nipponica* possesses 4–8 leaflets. *V. unijuga*, *V. unijuga* var. *kaussanensis*, *V. linearifolia*, and *V. unijuga* f. *angustifolia* are known to be related taxa, and all possess two leaflets (Lee, 1972; Seok and Choi, 1997). *V. unijuga* var. *kaussanensis*, found on Jeju Island in South Korea, has overall smaller leaflets than *V. unijuga*, and *V. unijuga* f. *angustifolia* has lanceolate-shaped leaves, which is distinguished from *V. unijuga* that has ovate leaflets. *V. linearifolia* has been distinguished from other *Vicia* taxa, as it has lanceolate shape, acuminate apex leaflets, and white flowers (Lee, 1972; Seok and Choi, 1997). In this study, seed morphological characteristics such as seed color (greenish brown), hilum color (dark), and seed mottling (absent), and an oblong seed shape can be additional characteristics to distinguish *V. linearifolia*.

Style shape and pubescence characteristics are known to be major classification characteristics in the *Vicia*, *Lathyrus*, *Lens*, *Pisum*, and *Vavilovia* genera. Most *Vicia* species are laterally compressed, terete, and dorsiventrally compressed in terms of style shape and have abaxial, V-shaped, and even style pubescence (Kupicha, 1976; Endo and Ohashi, 1995; Choi et al., 2006). In the present study, most *Vicia* species distributed in South Korea had dorsally compressed styles, with *V. cracca* and *V. villosa* possessing laterally compressed styles and a terete shape being observed in *V. hirsuta*, *V. dasycarpa*, and *V. amurensis*. *V. dasycarpa* of sect. *Cracca* has similar characteristics to *V. villosa*, and two are known to be closely related. The two taxa can be divided based on overall hair density; however, owing to the fact that they share very similar characteristics, *V*. *dasycarpa* is recognized as a subspecies or by synonyms of *V. villosa* (Jalilian et al., 2014; Jung et al., 2016). However, the newly investigated terete shape and abaxially tufted style of *V. dasycarpa* can be used to distinguish the species from *V. villosa*, which is laterally compressed and evenly pubescent in style (Table 2 and Supplementary Table S2). These characteristics could be utilized as a taxonomic key between the two taxa, allowing *V. dasycarpa* to be recognized as a species in its own right.

### Molecular and Morphological Analysis of South Korean *Vicia* Species

In the present study, results revealed that the combined barcoding regions (ITS2 + *matK* + *rbcL*) efficiently differentiate the following species: *V. angustifolia* var. *segetilis*, *V. bungei*, *V. villosa*, *V. cracca*, *V. dasycarpa*, *V. hirsuta*, *V. tetrasperma*, *V. amurensis*, *V. hirticalycina*, and *V. chosenensis*. However, they failed to differentiate the species of *V. unijuga*, *V. unijuga* var. *kaussanensis*, *V. linearifolia*, *V. unijuga* f. *angustifolia*, *V. nipponica*, *V. amoena*, *V. venosa* var. *cuspidata*, *V. pseudo-orobus*, and *V. japonica* with the tested barcode regions. To differentiate the unclassified species, 39 morphological characteristics were investigated, in which 16 useful characteristics such as tuber presence, stem pubescence, number of leaflets, leaflet shape, leaflet apex, leaflet base, stipule nectariferous spot, stipule presence, tendrils, number of flowers per inflorescence, relative length of limb and claw in standard, standard shape, seed shape, seed color, style shape, and ovary hairiness were selected for efficient classification. The 16 selected useful traits were efficiently differentiating the closely related species of *V. unijuga*, *V. unijuga* var. *kaussanensis, V. linearifolia*, *V. unijuga* f. *angustifolia*, *V. nipponica*, *V. amoena*, *V. venosa* var. *cuspidata*, *V. pseudo-orobus*, and *V. japonica* when analyzed with tested barcodes.

DNA barcoding and morphological characters have long been studied as efficient methods of species classification (Li et al., 2011; Ghorbani et al., 2017; Veldman et al., 2020). The present study also revealed that combination of DNA barcodes and morphological characterization were shown to be valuable methods in distinguishing among related species to classify the *Vicia* species found in South Korea. Most of the *Vicia* taxa collected in South Korea belonged to sect. *Vicilla* (12 taxa), sect. *Cracca* (three taxa), sect. *Vicia* (one taxon), sect. *Americanae* (one taxon), sect. *Ervum* (one taxon), and sect. *Lenticula* (one taxon). *V. nipponica*, *V. unijuga* f. *angustifolia*, *V. linearifolia*, *V. unijuga*, and *V. unijuga* var. *kaussanensis* H. Lév. of sect. *Vicilla* have almost the same nucleotide base composition and were more closely related among the examined taxa (Supplementary Figures S1–3). The shape and size of leaflets are important classification keys for *V. unijuga* f. *angustifolia*, *V. linearifolia*, *V. unijuga*, and *V. unijuga* var. *kaussanensis*; however, leaflet sizes are easily differentiated and easily influenced by the external environment (Seok and Choi, 1997). Thus, more stable characteristics such as number of leaflets, leaflet shape, stem pubescence, leaflet shape, seed shape, and seed color are needed to distinguish these species. *V. nipponica* possesses the very distinct characteristic of having four or more leaflets compared to *V. unijuga* f. *angustifolia*, *V. linearifolia*, *V. unijuga* var. *kaussanensis*, and *V. unijuga*, which only have two leaflets. However, the molecular phylogeny analysis showed that the species is genetically more related with other species.

In the case of closely related species or species that have recently undergone speciation, morphological characteristics are considered important because identification with barcode regions is difficult. The accumulation of genetic and ecological differences can occur because of geographical isolation and ecology (Ba̧czkiewicz et al., 2017). For example, in *V. japonica*, the accessions collected on Ulleung Island share the same nucleotide sequences in the barcode regions, whereas one specimen collected in Pohang showed variation. This is probably the result of geographic isolation, and further studies are needed to determine whether these regions have different genetic diversity. Moreover, a species with the highest level of intraspecific difference was observed with *V. venosa* var. *cuspidata*. The chromosome number of *V. venosa* var. *cuspidata* was found to be 2*n* = 4X = 24, also known as autotetrasperma (Nam et al., 2012). It was reported that in the ITS region, autotetrasperma plants sometimes have two types of nucleotide sequence, which may be the reason for the observed high intraspecific variation (Noh et al., 2018).

## Conclusion

In conclusion, based on the molecular phylogenetic analysis, the present study suggests that, within the *Vicia* species found in South Korea, combined barcode regions (ITS2, *matK*, and *rbcL*) could be applied to distinguish the 10 *Vicia* taxa such as *V. angustifolia* var. *segetilis*, *V. bungei*, *V. villosa*, *V. cracca*, *V. dasycarpa*, *V. hirsuta*, *V. tetrasperma*, *V. amurensis*, *V. hirticalycina*, and *V. chosenensis*, whereas nine *Vicia* taxa such as *V. unijuga*, *V. unijuga* var. *kaussanensis*, *V. linearifolia*, *V. unijuga* f. *angustifolia*, *V. nipponica*, *V. amoena*, *V. pseudo-orobus*, and *V. japonica* can only be effectively differentiated along with 16 morphologically useful character. Recommended candidate barcode regions for species discrimination could not distinguish all species native to South Korea; thus, further molecular studies on a new barcode locus or effective molecular markers from nuclear and chloroplast genomes are needed for the discrimination of closely related species or species that have recently undergone speciation in the genus *Vicia*. The results of molecular and morphological characteristics of this study could be a valuable information for the discrimination of *Vicia* species in agricultural, ecological, and conservation purpose.

## Data Availability Statement

The datasets generated in this study can be found in online repositories. All the sequences were deposited to NCBI GenBank with following accession numbers: MW372935-MW373052 and MW374737-MW374795.

## Author Contributions

DH and J-WC conceived and designed the experiments. SH, RS, and KL performed the experiments and analyzed the data. G-TC and DH contributed to project coordination and analysis. SH and XW contributed to materials. SH and RS drafted the manuscript and figures. All authors contributed to the article and approved the final version of the submitted manuscript.

## Conflict of Interest

The authors declare that the research was conducted in the absence of any commercial or financial relationships that could be construed as a potential conflict of interest.
