# Supplementary material for: Identification of Vicia Species Native to South Korea Using Molecular and Morphological Characteristics
Source: Front Plant Sci. 2021 Feb 9;12:608559. doi: 10.3389/fpls.2021.608559 (PMC7900155; doi:10.3389/fpls.2021.608559)
Supplement: Supplementary file 1 [file Presentation_1.PPTX]

## Slide 1
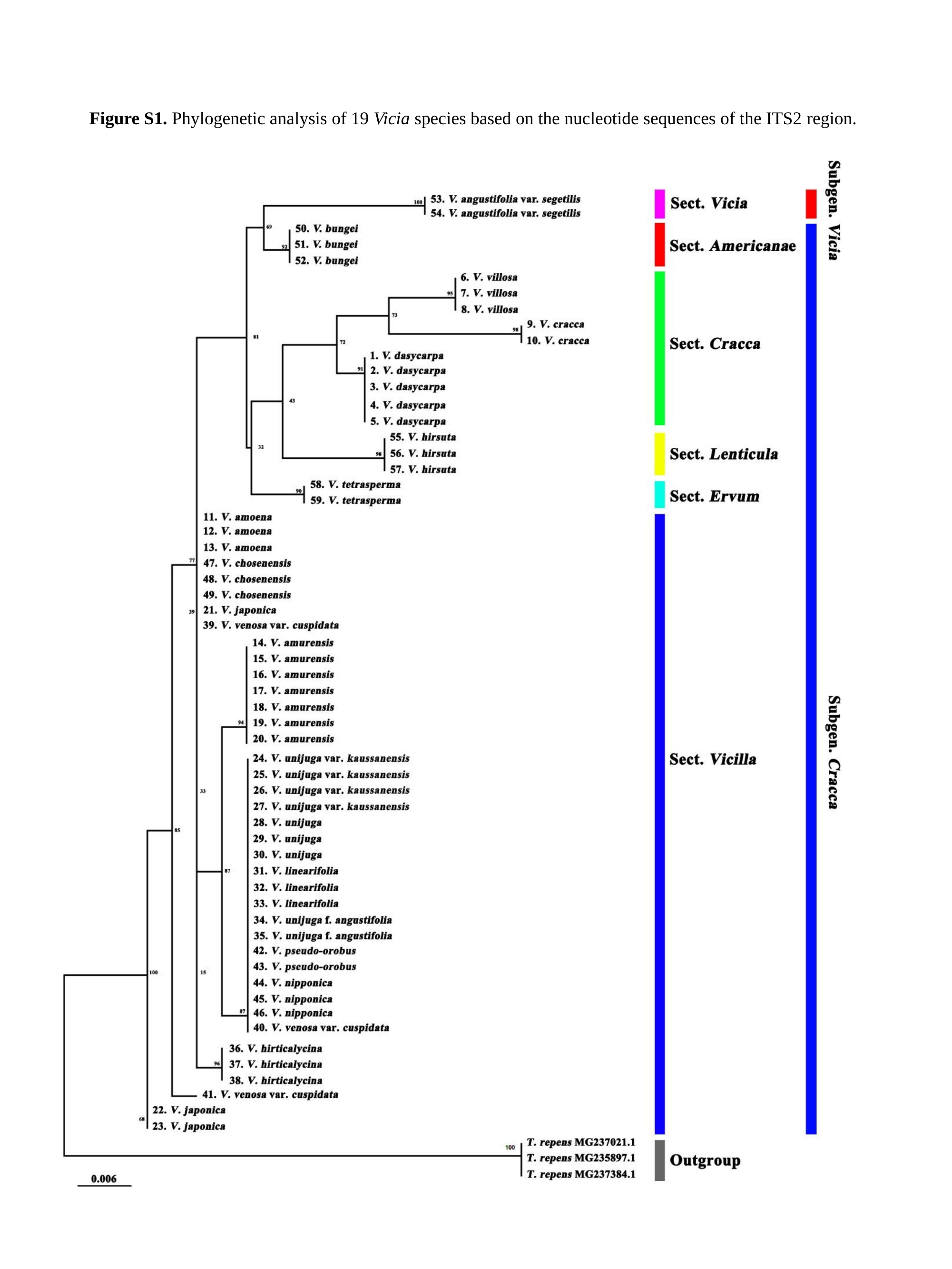

Figure S1. Phylogenetic analysis of 19 Vicia species based on the nucleotide sequences of the ITS2 region.

## Slide 2
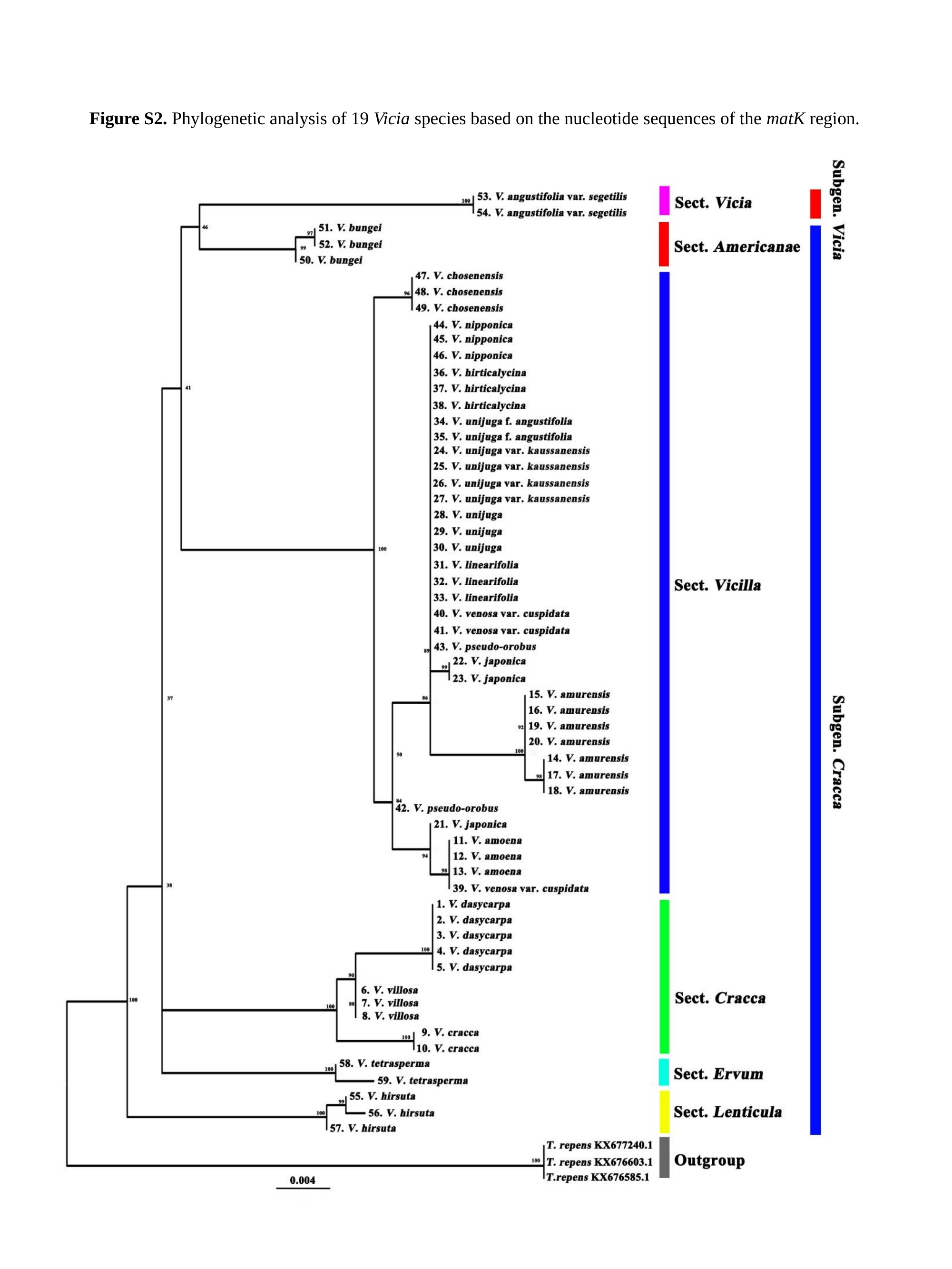

Figure S2. Phylogenetic analysis of 19 Vicia species based on the nucleotide sequences of the matK region.

## Slide 3
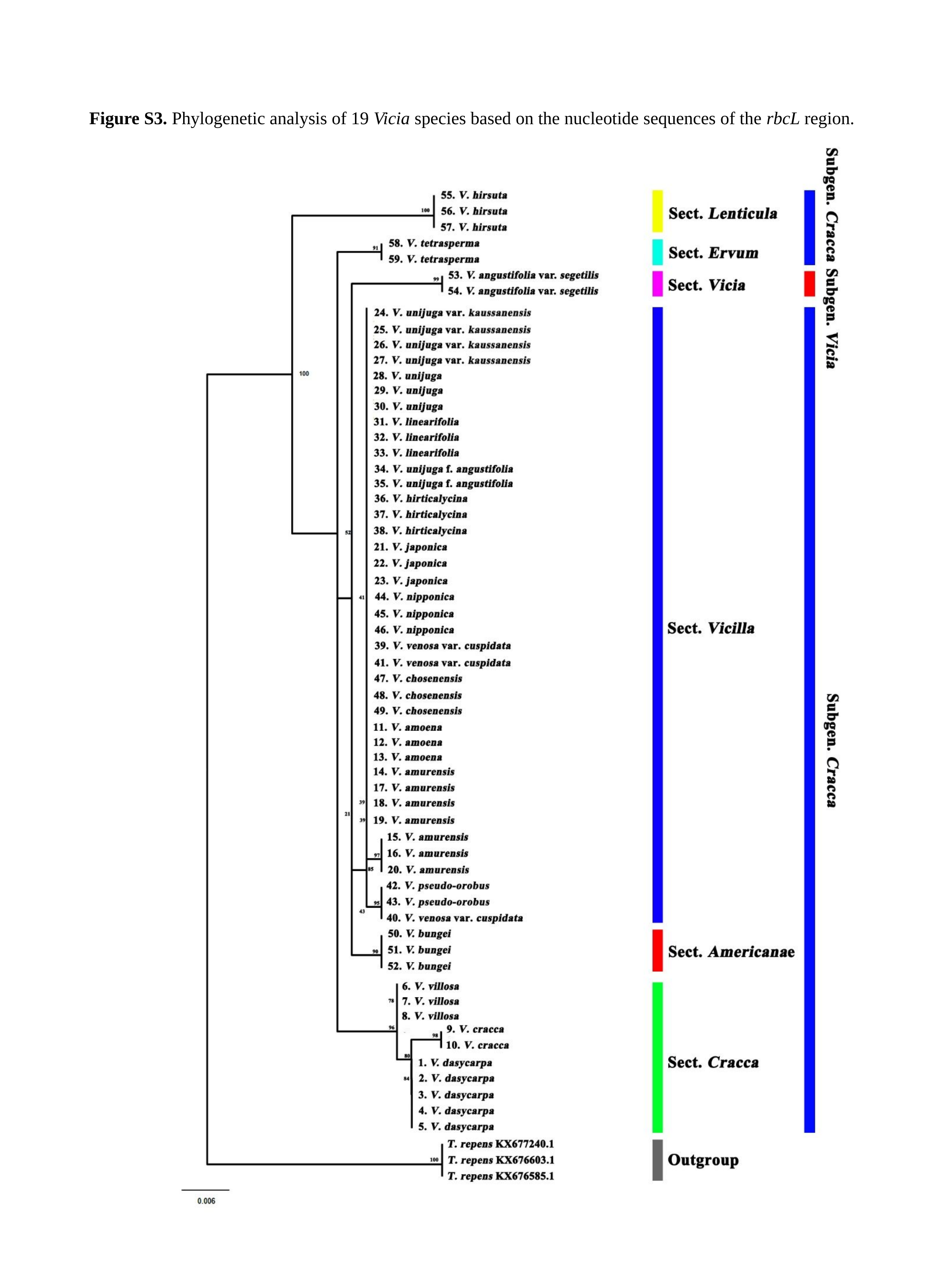

Figure S3. Phylogenetic analysis of 19 Vicia species based on the nucleotide sequences of the rbcL region.

## Slide 4
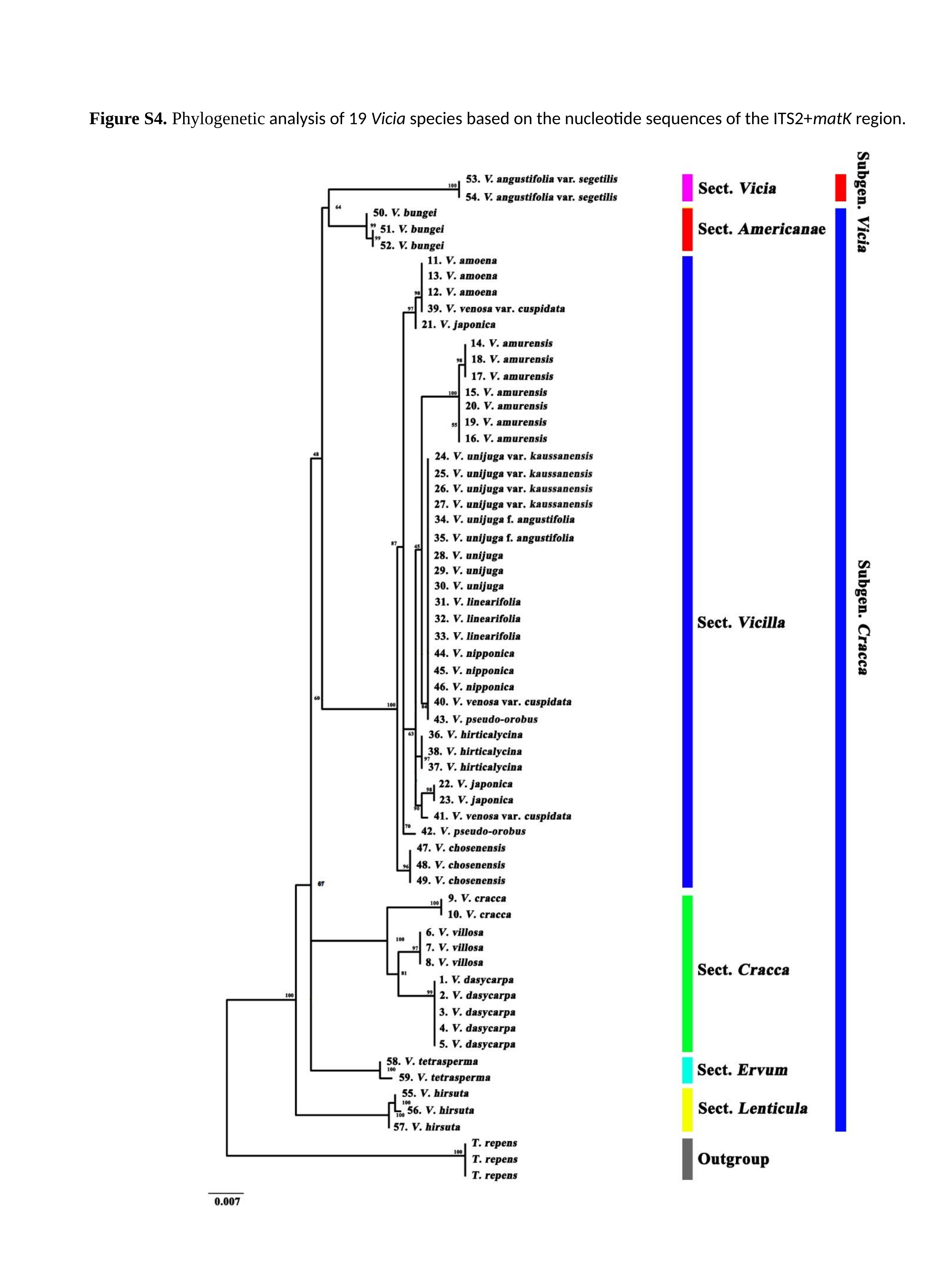

Figure S4. Phylogenetic analysis of 19 Vicia species based on the nucleotide sequences of the ITS2+matK region.

## Slide 5
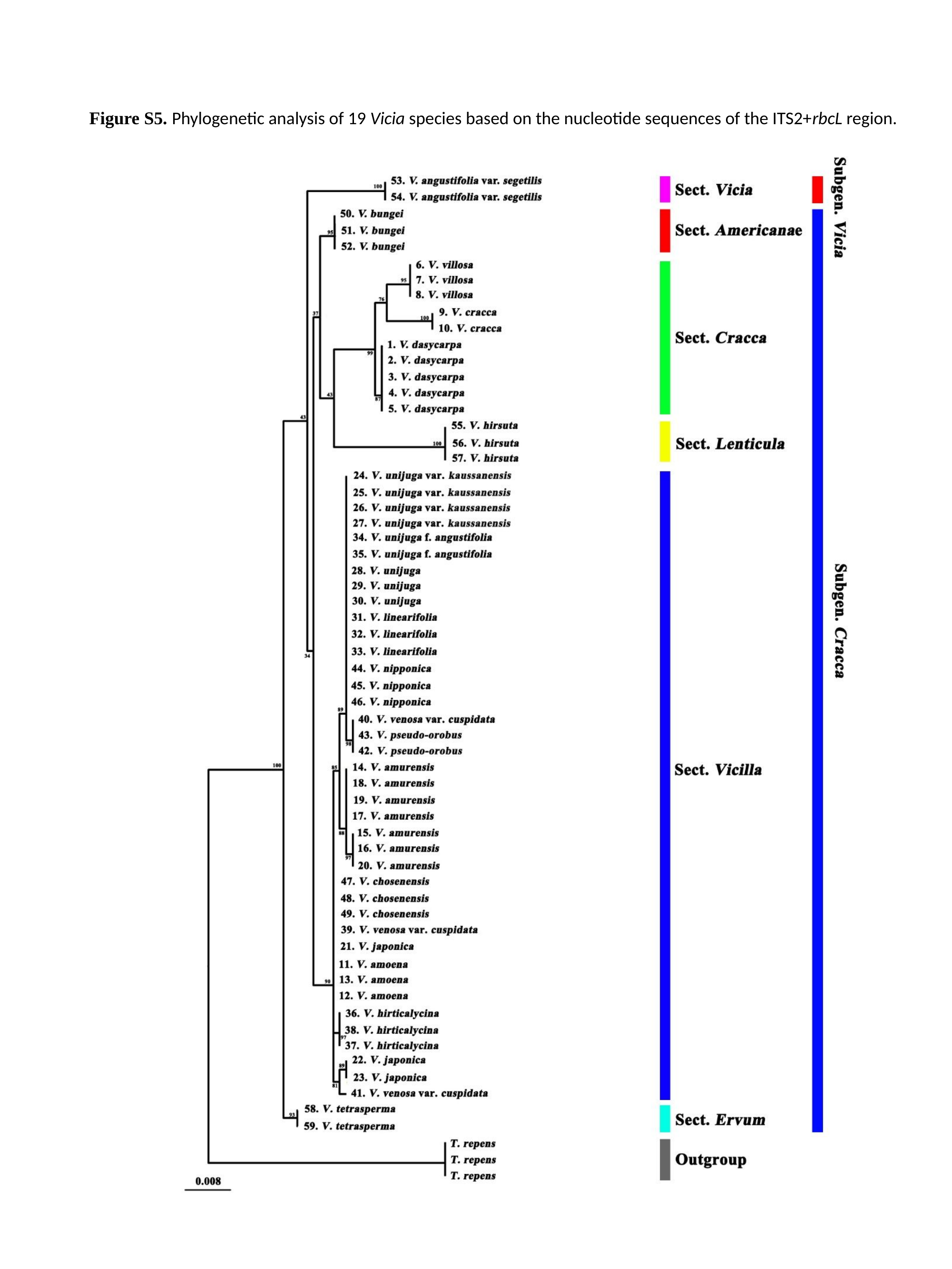

Figure S5. Phylogenetic analysis of 19 Vicia species based on the nucleotide sequences of the ITS2+rbcL region.

## Slide 6
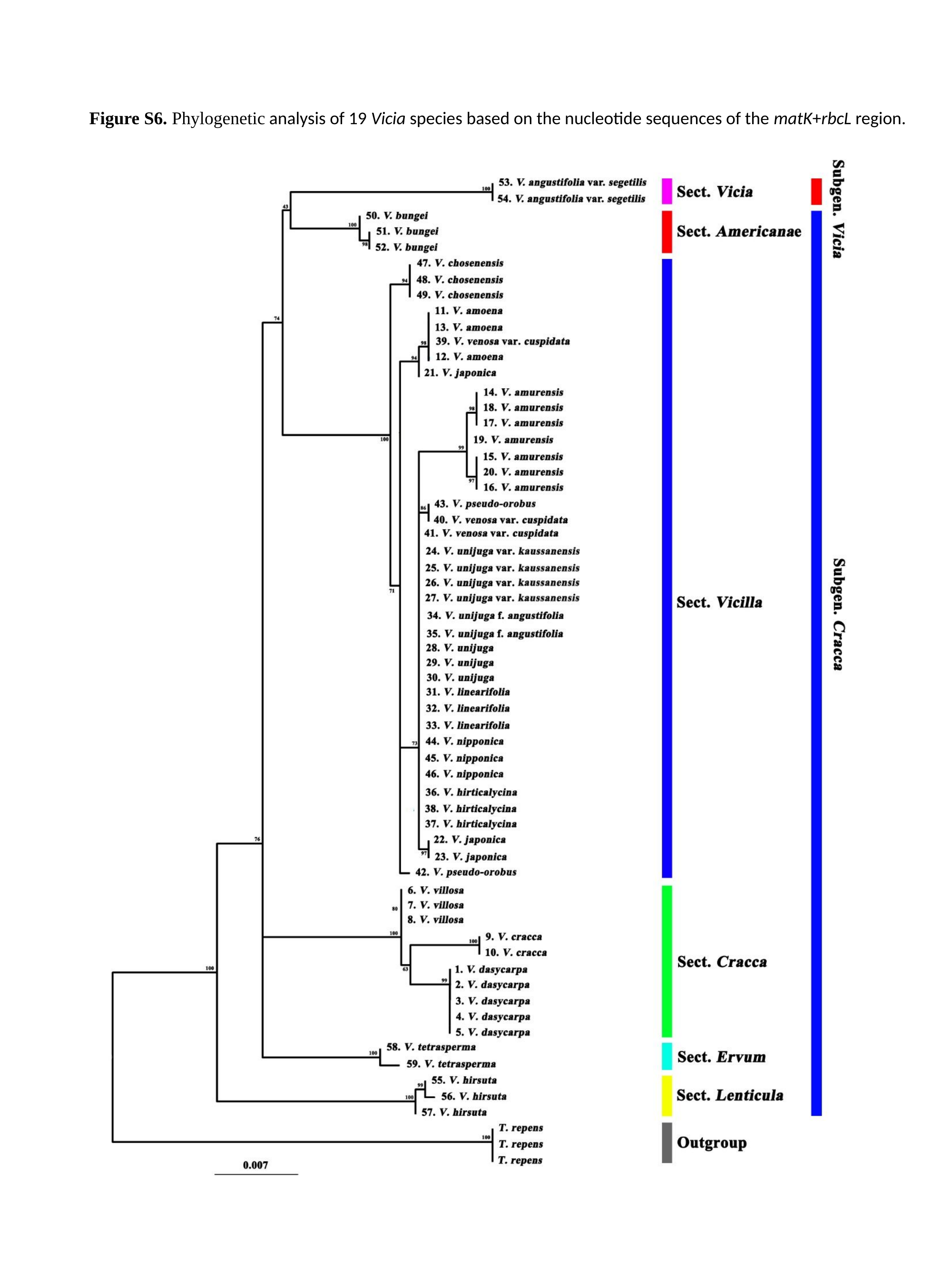

Figure S6. Phylogenetic analysis of 19 Vicia species based on the nucleotide sequences of the matK+rbcL region.
