# Supplementary material for: Identification of Vicia Species Native to South Korea Using Molecular and Morphological Characteristics
Source: Front Plant Sci. 2021 Feb 9;12:608559. doi: 10.3389/fpls.2021.608559 (PMC7900155; doi:10.3389/fpls.2021.608559)
Supplement: Supplementary file 4 [file Table_3.DOCX]

Supplementary Material

# Supplementary Data

## Supplementary Tables

**Table S1.** Information on accessions and barcoding sequences of *Vicia* from NCBI GenBank

**Table S2.** Species and morphological characteristics of *Vicia* species

## Supplementary Figures

**Figure S1.** Phylogenetic analysis of 19 *Vicia* species based on the nucleotide sequences of the ITS2 region.

**Figure S2.** Phylogenetic analysis of 19 *Vicia* species based on the nucleotide sequences of the *matK* region.

**Figure S3.** Phylogenetic analysis of 19 *Vicia* species based on the nucleotide sequences of the *rbcL* region.

**Figure S4.** Phylogenetic analysis of 19 *Vicia* species based on the nucleotide sequences of the combined ITS2+*matK* regions.

**Figure S5.** Phylogenetic analysis of 19 *Vicia* species based on the nucleotide sequences of the combined ITS2+*rbcL* regions.

**Figure S6.** Phylogenetic analysis of 19 *Vicia* species based on the nucleotide sequences of the combined *matK*+*rbcL* regions.
